# Adjuvant and post-recurrent treatment patterns in patients with resectable gastric cancer in Japan: a retrospective database cohort study

**DOI:** 10.1007/s10120-024-01501-w

**Published:** 2024-04-30

**Authors:** Takaki Yoshikawa, Yorifumi Kikko, Reina Makino, Yuya Kimijima, Eiji Nishiyama, Yuko Matsuda, Bruno Casaes Teixeira, Mariella Tejada, Robert Carroll, Shuichi Hironaka

**Affiliations:** 1 National Cancer Center Hospital, Tokyo, Japan; 2 Bristol Myers Squibb, Tokyo, Japan; 3 Ono Pharmaceutical Co., Ltd, Osaka, Japan; 4 Bristol Myers Squibb, Uxbridge, UK; 5 Bristol Myers Squibb, Princeton, New Jersey USA; 6 Kyorin University Faculty of Medicine, Tokyo, Japan; 7 Saitama Medical University International Medical Center, Saitama, Japan

**Keywords:** Chemotherapy, adjuvant, Cohort studies, Practice patterns, physicians’, Retrospective studies, Stomach neoplasms

## Abstract

**Background:**

This study examined temporal shifts in adjuvant therapy patterns in Japanese patients with resectable gastric cancer (GC) and treatment patterns of first-line and subsequent therapy among those with recurrent disease.

**Methods:**

This retrospective analysis of hospital-based administrative claims data (April 1, 2008 to March 31, 2022) included adults (aged ≥ 20 years) with GC who started adjuvant therapy on or after October 1, 2008 (adjuvant cohort) and patients in the adjuvant cohort with disease recurrence (recurrent cohort), further defined by the time to recurrence (≤ 180 or > 180 days after adjuvant therapy).

**Results:**

In the adjuvant cohort (n = 17,062), the most common regimen during October 2008–May 2016 was tegafur/gimeracil/oteracil potassium (S-1; 95.7%). As new standard adjuvant regimen options were established, adjuvant S-1 use decreased to 65.0% and fluoropyrimidine plus oxaliplatin or docetaxel plus S-1 use increased to 15.0% and 20.0%, respectively, in September 2019–March 2022. In the recurrent cohort with no history of trastuzumab/trastuzumab deruxtecan treatment (n = 1257), the most common first-line regimens were paclitaxel plus ramucirumab (34.0%), capecitabine plus oxaliplatin (CapeOX; 17.0%), and nab-paclitaxel plus ramucirumab (10.1%) in patients with early recurrence, and S-1 plus oxaliplatin (26.3%), S-1 plus cisplatin (15.3%), CapeOX (14.0%), S-1 (13.2%), and paclitaxel plus ramucirumab (10.8%) in those with late recurrence.

**Conclusions:**

This study demonstrated temporal shifts in adjuvant treatment patterns that followed the establishment of novel regimens, and confirmed that post-recurrent treatment patterns were consistent with the Japanese Gastric Cancer Association guideline recommendations.

**Supplementary Information:**

The online version contains supplementary material available at 10.1007/s10120-024-01501-w.

## Introduction

Gastric cancer (GC) is the third most common cause of cancer-related death in Japan, with approximately 41,100 GC-related deaths in 2022 [1]. In patients with advanced resectable GC, surgical resection with adequate lymphadenopathy and adjuvant chemotherapy is the main strategy in Japan [2]. This is based on data from the pivotal phase III ACTS-GC study in Japanese patients with pathological stage (pStage) II–III GC [3, 4]. The current Japan Gastric Cancer Association (JGCA) guidelines recommend tegafur/gimeracil/oteracil potassium (S-1) for pStage II, and combination regimens including capecitabine plus oxaliplatin (CapeOX), S-1 plus oxaliplatin (SOX) and S-1 plus docetaxel (DS) as adjuvant therapy for pStage II–III disease [2], based on data from key phase III studies [5–7]. However, as direct comparisons of these regimens are limited, there is no clear guidance on how to select the most appropriate adjuvant therapy.

Despite advances in clinical outcomes with surgery plus adjuvant therapy, patients with recurrent tumors after adjuvant therapy typically have a poor prognosis [8, 9]. In particular, the optimal treatment strategy for patients with early recurrence is an important clinical question. In a retrospective study by Shitara and colleagues of 52 patients with recurrent GC after S-1 adjuvant therapy, patients with a recurrence-free interval of < 6 months had poorer treatment outcomes with post-recurrent first-line S-1 plus cisplatin (SP) than those with a recurrence-free interval of ≥ 6 months [10]. Therefore, the 2018 JGCA guidelines recommended not using chemotherapy drugs that had already been administered as adjuvant therapy to treat recurrent disease during or within 6 months of completing adjuvant chemotherapy [11]. However, the optimal strategy for patients with early recurrence is not established as most randomized clinical trials evaluating first-line chemotherapy have excluded these patients [12, 13].

The aging population in Japan has led to an increased number of GC-related deaths, as GC is more common in older individuals. Among 124,319 Japanese people with GC in 2019, those aged 65–74 years or ≥ 75 years accounted for approximately 32% and 53% of cases, respectively [14]. However, as the current guidelines for GC management are mainly based on evidence from randomized clinical trials conducted in patients aged < 75 years [15], the optimal strategy for older patients with GC is another important clinical question.

Development of large-scale administrative claims databases in Japan has allowed for assessment of real-world treatment patterns and outcomes [16]. A previous database study by Komatsu and colleagues reported on treatment patterns among Japanese patients with advanced or recurrent GC, identifying factors that were associated with overall therapy duration that may help to understand the optimal treatment sequence in these patients [17].

In this database study, the main objectives were to examine temporal shifts in treatment patterns for adjuvant therapy in Japanese patients with GC, and to describe treatment patterns of first-line and subsequent therapy among those with disease recurrence during or after adjuvant therapy according to the time to recurrence (i.e., early or late recurrence) and by age in the real-world setting.

## Methods

### Study design

This retrospective, observational, multicenter study used data extracted from a hospital-based administrative claims database provided by Medical Data Vision (MDV) Co., Ltd. (Tokyo, Japan). The MDV database contains de-identified health insurance claims data, collected from hospitals in Japan that use the Diagnostic Procedure combination (DPC) data collection system for acute inpatient care [18]. As of May 31, 2022, the database contained data from over 40 million patients including in- and out-patient claims for age, sex, diagnoses, medications, medical procedures, and information regarding the treatment hospital, as well as clinical information from discharge summaries.

This study used patient data from April 1, 2008 to March 31, 2022 (Supplementary Fig. S1). The claims codes for diagnoses, medical procedures, and medications used in this study are provided in Supplementary Tables S1 and S2.

### Study population

The adjuvant cohort included all patients with an initial diagnosis of GC who underwent surgical resection for GC within 60 days of initial diagnosis, received JGCA guideline-recommended adjuvant therapy within 90 days of last surgical resection, and were aged ≥ 20 years at the start of adjuvant therapy. Patients with a prescription record for any JGCA-listed medications other than adjuvant therapy within 90 days after surgery, a record of recurrence on or before last dose of adjuvant therapy, who received any antitumor agents not recommended by the JGCA guidelines before surgery, or who participated in a clinical trial after surgery were excluded. Adjuvant therapy patterns were assessed across three treatment periods based on the adjuvant therapy start date: (1) May 1, 2008 to May 31, 2016; (2) June 1, 2016 to August 31, 2019; and (3) September 1, 2019 to March 31, 2022 (Supplementary methods).

The recurrent cohort included all patients in the adjuvant cohort with a record of recurrence after the last prescription of adjuvant therapy who started a JGCA guideline-recommended or conditionally recommended first- or second-line regimen as post-recurrent first-line therapy between May 1, 2014 and November 24, 2021. Patients who had ≤ 21 days of prescription record for adjuvant agents were excluded. The recurrent cohort was further defined as those who started first-line therapy during or ≤ 180 days after adjuvant therapy (i.e., early recurrence) or > 180 days after the end of adjuvant therapy (i.e., late recurrence).

### Treatment definitions

The regimen in each line of treatment was defined as any combination of JGCA guideline-recommended or conditionally recommended antitumor agents prescribed within 90 days of treatment start date, except for trastuzumab (T-mab) or trastuzumab deruxtecan (T-DXd). The start date for each regimen was defined as the first date of administration of any agent in the regimen. If T-mab/T-DXd was added after the first 90 days and before the last administration of first-line or subsequent therapy, it was considered part of the corresponding first-line or subsequent regimen, respectively. Each line of treatment was considered ended when all antitumor agents in the regimen were not prescribed for 120 days after last administration or a new agent was started; if there was ≤ 120 days between the last administration of the agent(s) and any last record, such agent(s) were considered as continued and thus treated as censored at the last administration. Description of the index regimens for each line of treatment is provided in the Supplementary methods.

### Measures

Patient characteristics and hospital information data were extracted for the adjuvant and recurrent cohorts based on the start of adjuvant and first-line treatment, respectively. Data on selected comorbidities and metastasis site during the 180-day period before the start of adjuvant and first-line treatment were collected for the adjuvant and recurrent cohorts, respectively.

The 10-item Barthel Activities of Daily Living (ADL) index score was extracted to assess functional independence [19]. ADL index scores were defined as “independent” if all 10 items were reported as independent and “dependent” if any of the 10 items were reported as dependent.

Assessment of post-recurrent first-line or subsequent therapy was conducted separately based on whether or not patients had a history of T-mab/T-DXd treatment in any line (i.e., T-mab/T-DXd [ −] or T-mab/T-DXd [ +] groups). In the adjuvant and recurrent cohorts, an additional analysis by age category (< 65, 65–74, and ≥ 75 years) was conducted.

### Statistical analysis

Patient characteristics and hospital information were presented using descriptive statistics, with categorical variables presented as patient number and percentage, and continuous variables presented as median and range.

The median (95% confidence interval [CI]) duration of adjuvant and first-line therapy (from the first administration to the last administration or death, whichever came first) for the adjuvant and recurrent cohorts, as well as overall duration of therapy (from the first administration of post-recurrent first-line therapy to the last administration of the last line of therapy or death, whichever came first) for the recurrent cohort were estimated using the Kaplan–Meier method. Additional analyses of post-recurrent first-line therapy and overall treatment durations by when first-line therapy was started (before or after October 2015; i.e., when ramucirumab-containing regimens were included in the JGCA guidelines [11]) and by age category were also conducted in the T-mab/T-DXd [ −] group.

Two sensitivity analyses were conducted for treatment patterns in the recurrent cohort. In the first analysis, a different threshold was used to define early and late recurrence (210 vs 180 days after the last administration of adjuvant therapy). In the second analysis, a different threshold was used to define the end of treatment (90 vs 120 days after final administration).

Treatment sequences for the recurrent cohort were visualized using Sankey flow diagrams [20]. Statistical analyses were undertaken with SAS version 9.4 (SAS Institute, Cary, NC, USA).

## Results

### Study population

**Fig. 1:**
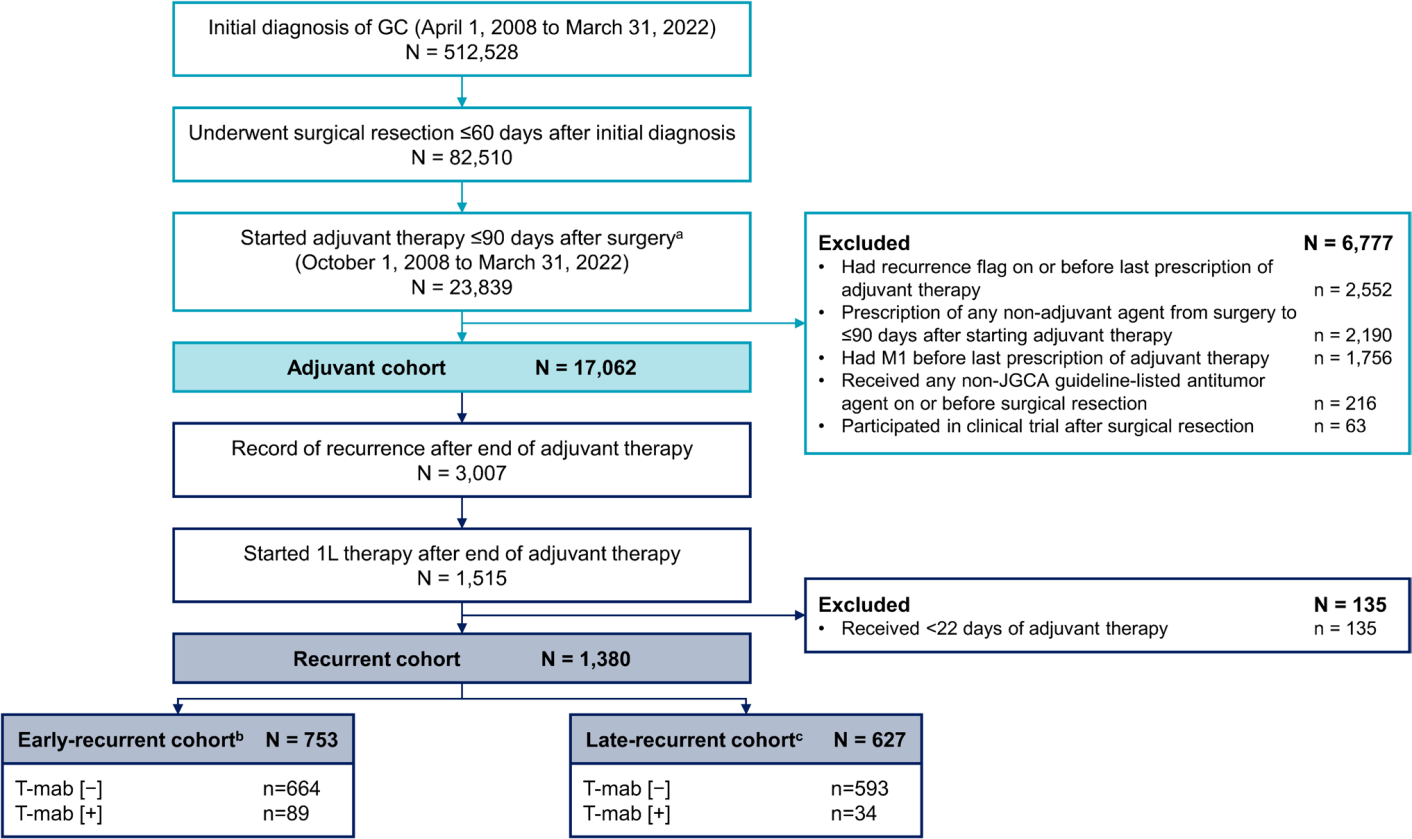
Patient flow. *1L* first-line, *GC* gastric cancer, *JGCA* Japanese Gastric Cancer Association, T-mab/T-DXd [ − ] with no history of trastuzumab or trastuzumab deruxtecan treatment, T-mab/T-DXd [ + ] with a history of trastuzumab or trastuzumab deruxtecan treatment. ^a^Aged ≥ 20 years at start of adjuvant therapy; ^b^Started post-recurrent 1L therapy ≤ 180 days after end of adjuvant therapy; ^c^Started post-recurrent 1L therapy > 180 days after end of adjuvant therapy

During the study period, 512,528 patients had a confirmed GC diagnosis, of whom 82,510 underwent surgical resection within 60 days of the diagnosis (Fig. 1). In total, 17,062 patients aged ≥ 20 years started JGCA guideline-directed adjuvant therapy on or after October 1, 2008 and were included in the adjuvant cohort. Of 3007 patients with recurrence after the end of adjuvant therapy, 1380 patients were included in the recurrent cohort; 753 patients had early recurrence and 627 patients had late recurrence.

Demographic and hospital information for patients were generally similar across the three age groups in the adjuvant cohort (Table 1) and in the T-mab/T-DXd [ − ] group of the recurrent cohort (Table 2). However, the prevalence of some comorbidities tended to be higher in patients aged ≥ 75 years than in younger patients in both cohorts. In the T-mab/T-DXd [ − ] group of recurrent cohort, the most common metastasis site was peritoneal or ascites, with this proportion being higher in the late versus early recurrent cohort. The demographic and hospital information for patients in the T-mab/T-DXd [ +] group of the recurrent cohort are summarized in Supplementary Table S3.

**Table 1:** Patient demographics and hospital admission information in the adjuvant cohort

Adjuvant cohort	All patients (N = 17,062)	Age group		
		< 65 years (n = 4826)	65–74 years (n = 7249)	≥ 75 years (n = 4987)
Male, n (%)	11,773 (69.0)	3202 (66.3)	5207 (71.8)	3364 (67.5)
Age,^a^ years, median (range)	70 (21–95)	58 (21–64)	70 (65–74)	78 (75–95)
Comorbidities,^b^ n (%)				
Hypertension	5388 (31.6)	923 (19.1)	2373 (32.7)	2092 (42.0)
Diabetes	4069 (23.9)	836 (17.3)	1885 (26.0)	1348 (27.0)
Liver disease	2119 (12.4)	591 (12.2)	924 (12.7)	604 (12.1)
Ischemic heart disease	1621 (9.5)	278 (5.8)	722 (10.0)	621 (12.5)
Thrombosis	1201 (7.0)	200 (4.1)	522 (7.2)	479 (9.6)
Kidney disease	628 (3.7)	119 (2.5)	261 (3.6)	248 (5.0)
Hemorrhoids	377 (2.2)	92 (1.9)	173 (2.4)	112 (2.2)
Neuropathy	373 (2.2)	76 (1.6)	152 (2.1)	145 (2.9)
Edema	300 (1.8)	54 (1.1)	130 (1.8)	116 (2.3)
ADL index score, n (%)				
Dependent	1842 (10.8)	264 (5.5)	710 (9.8)	868 (17.4)
Independent	7786 (45.6)	2176 (45.1)	3474 (47.9)	2136 (42.8)
Missing/incomplete	7434 (43.6)	2386 (49.4)	3065 (42.3)	1983 (39.8)
Designated cancer hospital,^b^ n (%)	13,540 (79.4)	3953 (81.9)	5798 (80.0)	3789 (76.0)
Department,^c^ n (%)				
Internal medicine^d^	864 (5.1)	232 (4.8)	395 (5.4)	237 (4.8)
Surgery^e^	15,678 (91.9)	4377 (90.7)	6683 (92.2)	4618 (92.6)
Other or unknown	1602 (9.4)	457 (9.5)	653 (9.0)	492 (9.9)
Number of beds in hospital, n (%)				
< 200	842 (4.9)	215 (4.5)	341 (4.7)	286 (5.7)
≥ 200 to < 500	9157 (53.7)	2557 (53.0)	3855 (53.2)	2745 (55.0)
≥ 500	7063 (41.4)	2054 (42.6)	3053 (42.1)	1956 (39.2)

*ADL* Activities of Daily Living

^a^At start of adjuvant first-line therapy

^b^In the 180 days prior to the start of adjuvant first-line therapy

^c^Patients may have multiple medical departments recorded

^d^Includes internal medicine, rheumatology and collagen disease internal medicine, and gastroenterology internal medicine departments

^e^Includes breast and thyroid, cardiovascular, neurosurgery, pediatric, hepato-biliary-pancreatic, cosmetic, dental-oral, general, respiratory, gastroenterological, and plastic surgery departments

**Table 2:** Patient demographics and hospital admission information in the recurrent cohort among patients in the T-mab/T-DXd [−] group

Recurrent T-mab/T-DXd [ −] cohort	All patients (N = 1257)	Early recurrence^a^ (N = 664)				Late recurrence^b^ (N = 593)			
		All patients (n = 664)	< 65 years (n = 147)	65–74 years (n = 305)	≥ 75 years (n = 212)	All patients (n = 593)	< 65 years (n = 168)	65–74 years (n = 265)	≥ 75 years (n = 160)
Male, n (%)	851 (67.7)	472 (71.1)	104 (70.7)	215 (70.5)	153 (72.2)	379 (63.9)	106 (63.1)	165 (62.3)	108 (67.5)
Age,^c^ years, median (range)	70 (28–90)	70 (31–90)	58 (31–64)	70 (65–74)	78 (75–90)	70 (28–89)	58 (28–64)	70 (65–74)	78 (75–89)
Comorbidities,^d^ n (%)									
Hypertension	196 (15.6)	111 (16.7)	15 (10.2)	54 (17.7)	42 (19.8)	85 (14.3)	19 (11.3)	36 (13.6)	30 (18.8)
Diabetes	116 (9.2)	68 (10.2)	9 (6.1)	33 (10.8)	26 (12.3)	48 (8.1)	13 (7.7)	26 (9.8)	9 (5.6)
Liver disease	139 (11.1)	83 (12.5)	15 (10.2)	40 (13.1)	28 (13.2)	56 (9.4)	18 (10.7)	20 (7.5)	18 (11.3)
Ischemic heart disease	38 (3.0)	21 (3.2)	2 (1.4)	11 (3.6)	8 (3.8)	17 (2.9)	6 (3.6)	5 (1.9)	6 (3.8)
Thrombosis	61 (4.9)	34 (5.1)	7 (4.8)	13 (4.3)	14 (6.6)	27 (4.6)	6 (3.6)	14 (5.3)	7 (4.4)
Kidney disease	145 (11.5)	58 (8.7)	16 (10.9)	23 (7.5)	19 (9.0)	87 (14.7)	28 (16.7)	39 (14.7)	20 (12.5)
Hemorrhoids	25 (2.0)	13 (2.0)	1 (0.7)	8 (2.6)	4 (1.9)	12 (2.0)	6 (3.6)	5 (1.9)	1 (0.6)
Neuropathy	73 (5.8)	45 (6.8)	15 (10.2)	20 (6.6)	10 (4.7)	28 (4.7)	9 (5.4)	13 (4.9)	6 (3.8)
Edema	56 (4.5)	38 (5.7)	3 (2.0)	19 (6.2)	16 (7.5)	18 (3.0)	6 (3.6)	8 (3.0)	4 (2.5)
ADL index, n (%)									
Dependent	54 (4.3)	32 (4.8)	7 (4.8)	13 (4.3)	12 (5.7)	22 (3.7)	6 (3.6)	9 (3.4)	7 (4.4)
Independent	811 (64.5)	426 (64.2)	95 (64.6)	199 (65.2)	132 (62.3)	385 (64.9)	112 (66.7)	176 (66.4)	97 (60.6)
Missing/incomplete	392 (31.2)	206 (31.0)	45 (30.6)	93 (30.5)	68 (32.1)	186 (31.4)	50 (29.8)	80 (30.2)	56 (35.0)
Metastasis site, n (%)									
Peritoneal (or ascites)	378 (30.1)	172 (25.9)	44 (29.9)	81 (26.6)	47 (22.2)	206 (34.7)	68 (40.5)	100 (37.7)	38 (23.8)
Lymph node	115 (9.1)	68 (10.2)	8 (5.4)	32 (10.5)	28 (13.2)	47 (7.9)	16 (9.5)	16 (6.0)	15 (9.4)
Liver	100 (8.0)	65 (9.8)	10 (6.8)	22 (7.2)	33 (15.6)	35 (5.9)	9 (5.4)	16 (6.0)	10 (6.3)
Lung	85 (6.8)	31 (4.7)	4 (2.7)	15 (4.9)	12 (5.7)	54 (9.1)	13 (7.7)	20 (7.5)	21 (13.1)
Bone	51 (4.1)	11 (1.7)	6 (4.1)	5 (1.6)	0	40 (6.7)	12 (7.1)	20 (7.5)	8 (5.0)
Brain	5 (0.4)	0	0	0	0	5 (0.8)	2 (1.2)	2 (0.8)	1 (0.6)
Designated cancer hospital,^d^ n (%)	992 (78.9)	515 (77.6)	121 (82.3)	237 (77.7)	157 (74.1)	477 (80.4)	142 (84.5)	216 (81.5)	119 (74.4)
Department,^e^ n (%)									
Internal medicine^f^	171 (13.6)	79 (11.9)	19 (12.9)	40 (13.1)	20 (9.4)	92 (15.5)	30 (17.9)	38 (14.3)	24 (15.0)
Surgery^g^	1012 (80.5)	562 (84.6)	125 (85.0)	254 (83.3)	183 (86.3)	450 (75.9)	120 (71.4)	201 (75.8)	129 (80.6)
Other or unknown	151 (12.0)	57 (8.6)	12 (8.2)	24 (7.9)	21 (9.9)	94 (15.9)	30 (17.9)	44 (16.6)	20 (12.5)
Number of beds in hospital, n (%)									
< 200	45 (3.6)	29 (4.4)	6 (4.1)	13 (4.3)	10 (4.7)	16 (2.7)	7 (4.2)	5 (1.9)	4 (2.5)
≥ 200 to < 500	739 (58.8)	377 (56.8)	77 (52.4)	179 (58.7)	121 (57.1)	362 (61.0)	99 (58.9)	157 (59.2)	106 (66.3)
≥ 500	473 (37.6)	258 (38.9)	64 (43.5)	113 (37.0)	81 (38.2)	215 (36.3)	62 (36.9)	103 (38.9)	50 (31.3)

*ADL* Activities of Daily Living, *T-mab/T-DXd [−]* with no history of trastuzumab or trastuzumab deruxtecan treatment

^a^Post-recurrent first-line therapy started ≤ 180 days after end of adjuvant therapy

^b^Post-recurrent first-line therapy started > 180 days after end of adjuvant therapy

^c^At start of first-line therapy post-recurrence

^d^In the 180 days prior to the start of post-recurrent first-line therapy

^e^Patients may have multiple medical departments recorded

^f^Includes internal medicine, rheumatology and collagen disease internal medicine, and gastroenterology internal medicine departments

^g^Includes breast and thyroid, cardiovascular, neurosurgery, pediatric, hepato-biliary-pancreatic, cosmetic, dental-oral, general surgery, respiratory, gastroenterological, and plastic surgery departments

### Adjuvant therapy patterns

**Fig. 2:**
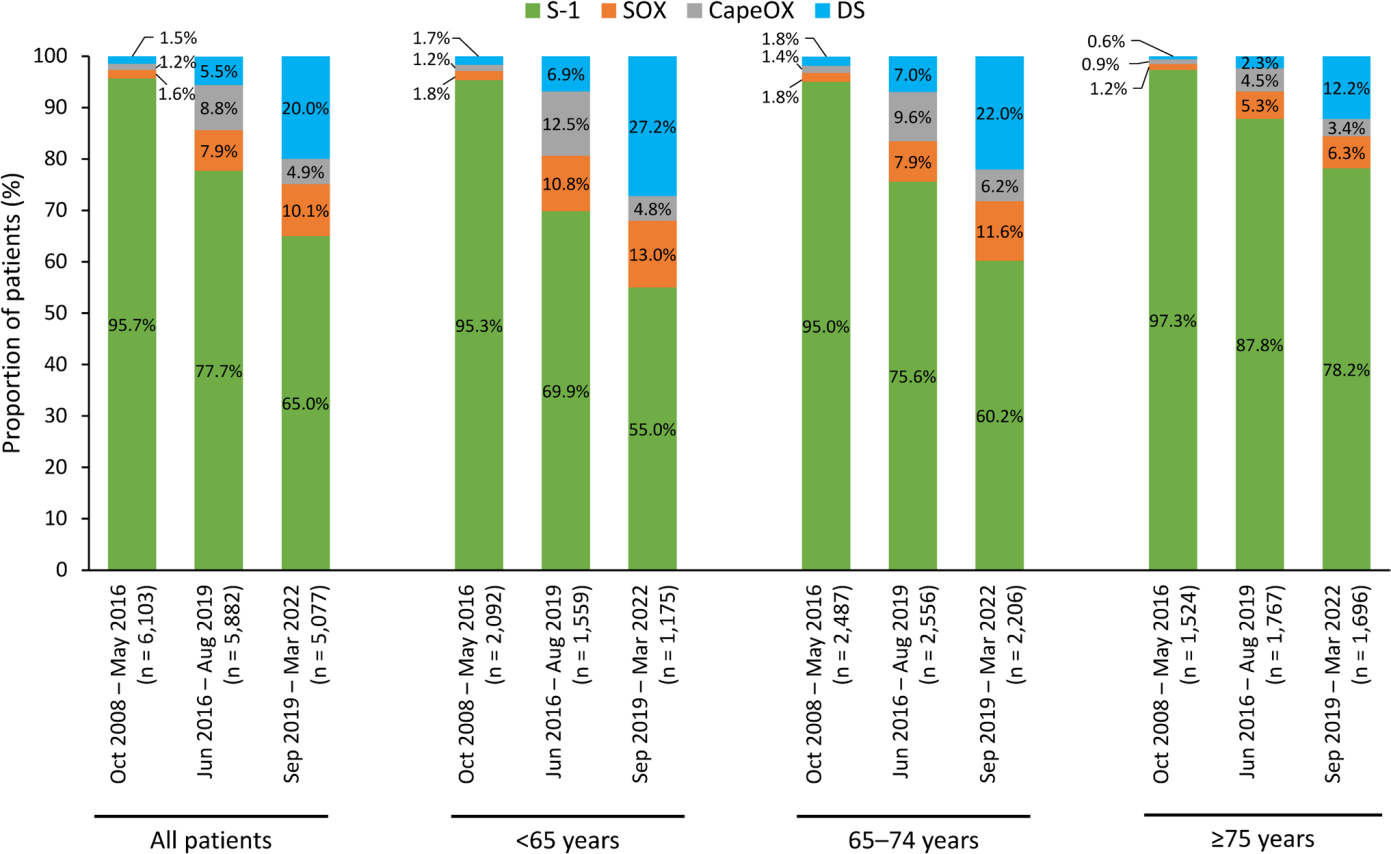
Adjuvant treatment patterns by time period in the adjuvant therapy cohort (N = 17,062). *CapeOX* capecitabine plus oxaliplatin, *DS* S-1 plus docetaxel, *S-1* tegafur/gimeracil/oteracil potassium, *SOX* S-1 plus oxaliplatin

Across all three time periods, the most common adjuvant therapy regimen was S-1 (Fig. 2). The proportion of patients treated with S-1 decreased over time from 95.7% during October 2008–May 2016 to 77.7% during June 2016–August 2019 and 65.0% during September 2019–March 2022. The proportion of patients treated with fluoropyrimidine plus oxaliplatin combination regimens increased from 2.8% during October 2008–May 2016 to 16.7% during June 2016–August 2019 and 15.0% during September 2019–March 2022 after these regimens were recommended by the JGCA guidelines in June 2016 [2]. The proportion of patients receiving DS adjuvant therapy also increased from 1.5% and 5.5% during October 2008–May 2016 and June 2016–August 2019, respectively, to 20.0% in September 2019–March 2022 after this regimen was recommended in the JGCA guidelines in September 2019.

The median duration of each adjuvant regimen generally remained similar across the three treatment periods, although SOX had a slightly lower median duration in the second time period versus the first and third periods (7.8 vs 10.0 and 10.2 months, respectively; Table 3).

**Table 3:** Duration of adjuvant therapy for gastric cancer according to adjuvant therapy start date in the adjuvant cohort (N = 17,062)

Treatment period	S-1 (n = 13,713)	SOX^b^ (n = 1077)	CapeOX^b^ (n = 843)	DS^c^ (n = 1429)	All regimens (N = 17,062)
October 1, 2008–May 31, 2016					
Patients, n (%)^c^	5840 (95.7)	100 (1.6)	73 (1.2)	90 (1.5)	6103 (100)
Median (95% CI) duration of therapy,^d^ months	10.6 (10.4–10.6)	10.0 (7.4–11.5)	5.7 (5.6–6.0)	10.9 (10.4–11.3)	10.5 (10.4–10.6)
June 1, 2016–August 31, 2019					
Patients, n (%)^c^	4573 (77.7)	463 (7.9)	520 (8.8)	326 (5.5)	5882 (100)
Median (95% CI) duration of therapy,^d^ months	10.5 (10.4–10.6)	7.8 (6.9–8.8)	5.6 (5.4–5.6)	11.1 (10.7–11.3)	10.1 (9.9–10.2)
September 1, 2019–March 31, 2022					
Patients, n (%)^c^	3300 (65.0)	514 (10.1)	250 (4.9)	1013 (20.0)	5077 (100)
Median (95% CI) duration of therapy,^d^ months	10.8 (10.8–11.0)	10.2 (8.3–11.1)	5.8 (5.6–6.0)	11.3 (11.3–11.5)	10.8 (10.8–11.0)

*CI* confidence interval, *CapeOX* capecitabine plus oxaliplatin, *DS* S-1 plus docetaxel, *JGCA* Japanese Gastric Cancer Association, *S-1* tegafur/gimeracil/oteracil potassium, *SOX* S-1 plus oxaliplatin

^a^Recommended as adjuvant therapy by JGCA guidelines since June 2016

^b^Recommended as adjuvant therapy by JGCA guidelines since September 2019

^c^Denominator based on patients treated with any adjuvant therapy during each treatment period

^d^Estimated by Kaplan–Meier method

During September 2019–March 2022, the proportion of patients who received S-1 was higher among patients aged ≥ 75 years (78.2%) than in those aged < 65 or 65–74 years (55.0% and 60.2%, respectively; Fig. 2). In contrast, the proportion of patients treated with SOX, CapeOX, or DS was lower in patients aged ≥ 75 years (6.3%, 3.4%, and 12.2%, respectively) than in those aged < 65 years (13.0%, 4.8%, and 27.2%, respectively) or 65–74 years (11.6%, 6.2%, and 22.0%, respectively).

### Post-recurrent treatment patterns

#### In the T-mab/T-DXd [ − ] group

**Fig. 3:**
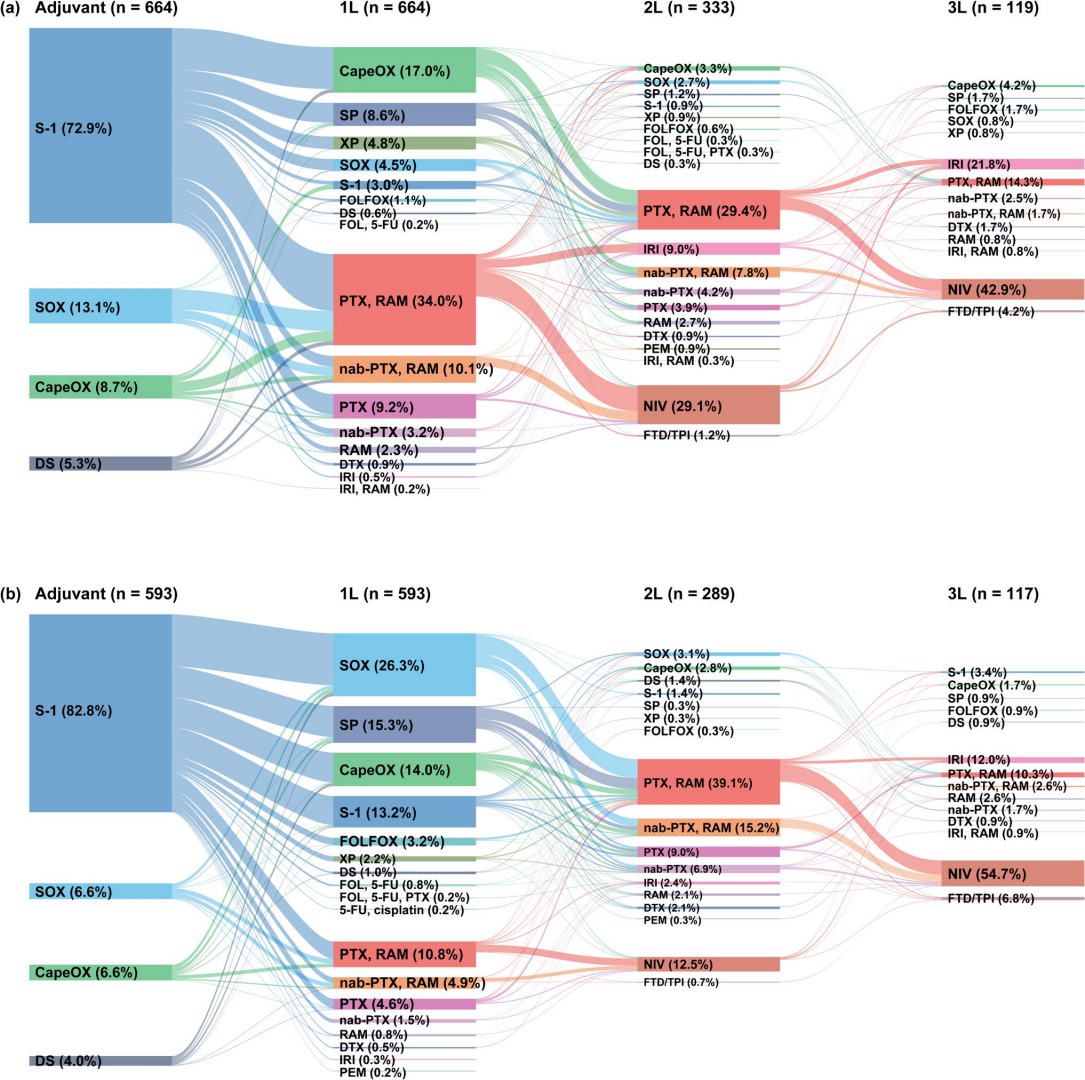
Treatment sequence in T-mab/T-DXd [ −] group of the recurrent cohort with **a** early recurrence (≤ 180 days from end of adjuvant therapy) or **b** late recurrence (> 180 days from end of adjuvant therapy). *1L* first-line, *2L* second-line, *3L* third-line, *5-FU* 5-fluorouracil, *CapeOX* capecitabine plus oxaliplatin, *DS* S-1 plus docetaxel, *DTX* docetaxel, *FLN* folinic acid, *FTD/TPI* trifluridine/tipiracil, *FOLFOX* folinic acid plus 5-fluorouracil plus oxaliplatin, *IRI* irinotecan, *nab-PTX* nab-paclitaxel, *NIV* nivolumab, *PEM* pembrolizumab, *PTX* paclitaxel, *RAM* ramucirumab, *S-1* tegafur/gimeracil/oteracil potassium, *SOX* S-1 plus oxaliplatin, *SP* S-1 plus cisplatin, T-mab/T-DXd [ −] with no history of trastuzumab or trastuzumab deruxtecan treatment, T-mab/T-DXd [ +] with a history of trastuzumab or trastuzumab deruxtecan treatment, *XP* capecitabine plus cisplatin

In the T-mab/T-DXd [ −] group of the early-recurrent cohort, the most common post-recurrent first-line regimens (in > 10% of patients) were paclitaxel plus ramucirumab (34.0%), CapeOX (17.0%), and nab-paclitaxel plus ramucirumab (10.1%; Fig. 3a, Table 4). Among patients who received these common regimens, a higher proportion of the patients who received paclitaxel or nab-paclitaxel plus ramucirumab had peritoneal metastasis or ascites at baseline compared with those who received CapeOX (31.4% and 35.8% vs 15.0%, respectively; Supplementary Table S4). In the late-recurrent cohort, the most common first-line regimens were SOX (26.3%), SP (15.3%), CapeOX (14.0%), S-1 (13.2%), and paclitaxel plus ramucirumab (10.8%; Fig. 3b, Table 4). In sensitivity analyses, first-line therapy patterns remained similar when early versus late recurrence was defined as first-line treatment started ≤ 210 versus > 210 days after adjuvant therapy (Supplementary Table S5) and when the definition of regimen end was defined as 90 days (Supplementary Table S6).

**Table 4:** Post-recurrent first-line treatment patterns in T-mab/T-DXd [ −] group of the recurrent cohort

	Early recurrence^a^ (n = 664)				Late recurrence^b^ (n = 593)			
	Patients (%)	1L treatment duration (months)^c^	1L → 2L transition (%)	Overall treatment duration (months)^c^	Patients (%)	1L treatment duration (months)^c^	1L → 2L transition (%)	Overall treatment duration (months)^c^
Total		4.6 (4.2–4.9)	55.9	9.0 (7.7–10.3)		4.9 (4.6–5.1)	55.2	9.5 (8.4–10.4)
With fluoropyrimidines								
CapeOX	113 (17.0)	4.6 (4.2–5.1)	69.8	11.9 (9.1–15.0)	83 (14.0)	4.9 (4.2–6.0)	58.7	9.7 (7.2–11.5)
SP	57 (8.6)	4.0 (2.9–5.0)	72.2	12.1 (7.5–18.2)	91 (15.3)	5.1 (4.3–6.1)	66.7	10.0 (8.1–14.3)
XP	32 (4.8)	4.5 (2.6–6.0)	68.0	12.9 (6.9–27.9)	13 (2.2)	3.5 (2.6–5.9)	61.5	9.3 (3.3–14.3)
SOX	30 (4.5)	4.3 (3.4–5.8)	64.3	7.5 (5.4–15.6)	156 (26.3)	5.1 (4.3–5.9)	60.4	10.5 (8.1–14.0)
S-1	20 (3.0)	4.9 (1.9–7.2)	65.0	17.3 (4.7–28.8)	78 (13.2)	4.9 (3.2–5.6)	38.2	9.0 (5.6–10.4)
FOLFOX	7 (1.1)	3.6 (1.0–7.3)	57.1	7.3 (1.4–NR)	19 (3.2)	4.6 (1.1–6.0)	46.7	13.4 (1.1–33.3)
DS	4 (0.6)	7.7 (6.2–12.5)	75.0	13.7 (6.2–NR)	6 (1.0)	5.6 (0.8–10.6)	40.0	10.0 (4.1–10.6)
FOL + 5-FU	1 (0.2)	1.2	100.0	3.1	5 (0.8)	2.8 (0.1–15.1)	60.0	5.0 (0.1–18.7)
FOL + 5-FU + cisplatin	0	–	–	–	1 (0.2)	1.1	100.0	42.3
FOL + 5-FU + PTX	0	–	–	–	1 (0.2)	3.4	100.0	NA^d^
Without fluoropyrimidines								
PTX + RAM	226 (34.0)	5.0 (4.6–5.6)	52.8	8.8 (7.2–10.9)	64 (10.8)	5.6 (3.9–6.8)	50.0	8.9 (5.9–12.3)
nab-PTX + RAM	67 (10.1)	4.9 (3.5–5.6)	44.8	6.5 (5.0–12.0)	29 (4.9)	5.1 (3.7–8.1)	52.2	8.9 (5.1–17.6)
PTX	61 (9.2)	3.4 (1.4–4.0)	30.8	3.5 (1.4–7.3)	27 (4.6)	3.7 (2.4–6.2)	52.0	5.6 (2.4–11.5)
nab-PTX	21 (3.2)	2.8 (2.1–4.1)	33.3	4.2 (2.1–9.3)	9 (1.5)	4.1 (1.4–13.6)	42.9	5.8 (1.4–13.9)
RAM	15 (2.3)	4.0 (0.1–5.3)	40.0	6.7 (0.1–10.6)	5 (0.8)	2.6 (0.1–3.3)	25.0	3.3 (0.1–11.1)
DTX	6 (0.9)	3.4 (1.4–5.6)	60.0	7.4 (1.4–10.7)	3 (0.5)	4.9 (4.7–34.4)	33.3	6.6 (4.9–34.4)
IRI	3 (0.5)	3.4 (1.4–8.8)	66.7	13.7 (1.4–36.3)	2 (0.3)	NR (2.6-NR)	0.0	NR (2.6-NR)
IRI + RAM	1 (0.2)	3.5	0.0	3.5	0	–	–	–
PEM	0	–	–	–	1 (0.2)	0.1	0.0	0.1

*1L* first line, *2L* second line, *5-FU* 5-fluorouracil, *CapeOX* capecitabine plus oxaliplatin, *DTX* docetaxel, *DS* S-1 plus docetaxel, *FOL* folinic acid, *FOLFOX* folinic acid plus 5-fluorouracil plus oxaliplatin, *IRI* irinotecan, *NA* not available, *nab-PTX* nab-paclitaxel, *NR* not reached, *PEM* pembrolizumab, *PTX* paclitaxel, *RAM* ramucirumab, *S-1* tegafur/gimeracil/oteracil potassium, *SOX* S-1 plus oxaliplatin, *SP* S-1 plus cisplatin, T-mab/T-DXd [ −] with no history of trastuzumab or trastuzumab deruxtecan treatment, *XP* capecitabine plus cisplatin

^a^Post-recurrent first-line therapy started ≤ 180 days after end of adjuvant therapy

^b^Post-recurrent first-line therapy started > 180 days after end of adjuvant therapy

^c^Data presented as median (95% confidence interval)

^d^Patient was censored after 2L therapy start

In the early-recurrent cohort, 596 patients discontinued first-line therapy or died, of whom 333 (55.9%) received second-line therapy (Fig. 3a). The most common second-line regimens were paclitaxel plus ramucirumab (29.4%) and nivolumab (29.1%). Among 119 patients who received third-line therapy, the most common regimens were nivolumab (42.9%), irinotecan (21.8%), and paclitaxel plus ramucirumab (14.3%).

In the late-recurrent cohort, 524 patients discontinued first-line therapy or died, of whom 289 (55.2%) received second-line therapy (Fig. 3b). The most common second-line regimens were paclitaxel plus ramucirumab (39.1%), nab-paclitaxel plus ramucirumab (15.2%), and nivolumab (12.5%). Among patients who received third-line therapy (n = 117), the most common regimens were nivolumab (54.7%), irinotecan (12.0%), and paclitaxel plus ramucirumab (10.3%).

The median duration of first-line therapy was similar in the early- and late-recurrent cohorts (4.6 vs 4.9 months; Table 4). The overall treatment duration (from the start of first-line therapy to end of the last line of therapy) was also similar in patients with early and late recurrence (median 9.0 vs 9.5 months). The proportion of patients who received second-line therapy was 55.9% and 55.2% in the early- and late-recurrent cohorts, respectively. When treatment durations were assessed in patients who started before and after October 2015, patients who started first-line therapy before October 2015 had numerically shorter median durations of first-line therapy and overall treatment in the early-recurrent cohort versus the late-recurrent cohort (first-line therapy, 3.5 vs 4.9 months, respectively; overall treatment 7.6 vs 10.0 months, respectively). In patients who started first-line therapy after October 2015, the median treatment durations were similar between the early- and late-recurrent cohorts (first-line therapy, 4.6 vs 4.9 months, respectively; overall treatment, 9.3 vs 9.5 months, respectively; Supplementary Table S7).

In the early-recurrent cohort, the median duration of first-line therapy for the most common regimens (i.e., paclitaxel plus ramucirumab, CapeOX, and nab-paclitaxel plus ramucirumab) was similar (5.0, 4.6, and 4.9 months, respectively; Table 4). However, the median overall treatment duration was numerically longer with CapeOX (11.9 months) than with paclitaxel plus ramucirumab and nab-paclitaxel plus ramucirumab (8.8 and 6.5 months, respectively), and the rate of first- to second-line transition was numerically higher with CapeOX (69.8%) than with paclitaxel plus ramucirumab (52.8%) and nab-paclitaxel plus ramucirumab (44.8%).

In the late-recurrent cohort, the median duration of first-line therapy and overall treatment was similar among the most common regimens, ranging from 4.9 to 5.6 months and 8.9 to 10.5 months, respectively (Table 4). However, the rate of first- to second-line transition was numerically lower with S-1 (38.2%) and paclitaxel plus ramucirumab (50.0%) than with fluoropyrimidine plus platinum regimens (60.4% for SOX, 66.7% for SP, and 58.7% for CapeOX).

First-line treatment patterns in the three age groups were generally similar in the early- and late-recurrent cohorts, but in the late-recurrent cohort, the median overall treatment duration was shorter in patients aged ≥ 75 years than in those aged < 65 years or 65–74 years (7.5 vs 10.4 and 9.5 months, respectively; Supplementary Table S8). The proportion of patients receiving first-line S-1 therapy in the late-recurrent cohort was higher in older versus younger patients (22.5% vs 5.4% and 12.5%, respectively), while the proportion of these patients who received SOX was slightly lower in older versus younger patients (20.6% vs 26.2% and 29.8%, respectively; Table 5).

**Table 5:** Post-recurrence first-line treatment regimens in T-mab/T-DXd [ −] group of the recurrent cohort by age group

	Early recurrence^a^ (n = 664)			Late recurrence^b^ (n = 593)		
	< 65 years	65–74 years	≥ 75 years	< 65 years	65–74 years	≥ 75 years
With fluoropyrimidines, n (%)						
CapeOX	25 (17.0)	58 (19.0)	30 (14.2)	26 (15.5)	31 (11.7)	26 (16.3)
SP	17 (11.6)	22 (7.2)	18 (8.5)	36 (21.4)	34 (12.8)	21 (13.1)
XP	7 (4.8)	19 (6.2)	6 (2.8)	4 (2.4)	8 (3.0)	1 (0.6)
SOX	8 (5.4)	16 (5.3)	6 (2.8)	44 (26.2)	79 (29.8)	33 (20.6)
S-1	6 (4.1)	7 (2.3)	7 (3.3)	9 (5.4)	33 (12.5)	36 (22.5)
FOLFOX	1 (0.7)	4 (1.3)	2 (0.9)	7 (4.2)	9 (3.4)	3 (1.9)
DS	1 (0.7)	2 (0.7)	1 (0.5)	2 (1.2)	3 (1.1)	1 (0.6)
FOL + 5-FU	0	1 (0.3)	0	2 (1.2)	3 (1.1)	0
FOL + 5-FU + cisplatin	0	0	0	1 (0.6)	0	0
FOL + 5-FU + PTX	0	0	0	0	1 (0.4)	0
Without fluoropyrimidines, n (%)						
PTX + RAM	48 (32.7)	96 (31.5)	82 (38.7)	18 (10.7)	31 (11.7)	15 (9.4)
nab-PTX + RAM	14 (9.5)	33 (10.8)	20 (9.4)	8 (4.8)	15 (5.7)	6 (3.8)
PTX	9 (6.1)	26 (8.5)	26 (12.3)	6 (3.6)	11 (4.2)	10 (6.3)
nab-PTX	4 (2.7)	10 (3.3)	7 (3.3)	4 (2.4)	1 (0.4)	4 (2.5)
RAM	4 (2.7)	6 (2.0)	5 (2.4)	1 (0.6)	3 (1.1)	1 (0.6)
DTX	1 (0.7)	4 (1.3)	1 (0.5)	0	2 (0.8)	1 (0.6)
IRI	2 (1.4)	0	1 (0.5)	0	1 (0.4)	1 (0.6)
IRI + RAM	0	1 (0.3)	0	0	0	0
PEM	0	0	0	0	0	1 (0.6)

*5-FU* 5-fluorouracil, *CapeOX* capecitabine plus oxaliplatin, *DTX* docetaxel, *DS* S-1 plus docetaxel, *FOL* folinic acid, *FOLFOX* folinic acid plus 5-fluorouracil plus oxaliplatin, *IRI* irinotecan, *nab-PTX* nab-paclitaxel, *PEM* pembrolizumab, *PTX* paclitaxel, *RAM* ramucirumab, *S-1* tegafur/gimeracil/oteracil potassium, *SOX* S-1 plus oxaliplatin, *SP* S-1 plus cisplatin, T-mab/T-DXd [ −] with no history of trastuzumab or trastuzumab deruxtecan treatment, *XP* capecitabine plus cisplatin

^a^Post-recurrent first-line therapy started ≤ 180 days after end of adjuvant therapy

^b^Post-recurrent first-line therapy started > 180 days after end of adjuvant therapy

#### In the T-mab/T-DXd [ +] group

Among 89 patients with early recurrence in the T-mab/T-DXd [ +] group, 73 (82.0%) received T-mab as part of their first-line regimen (Supplementary Fig. S2a). The most common first-line regimens were capecitabine plus cisplatin (XP) plus T-mab (49.4%), CapeOX plus T-mab (16.9%), and paclitaxel plus T-mab (10.1%).

In the late-recurrent cohort, 27 of 34 patients (79.4%) received a T-mab-containing first-line regimen (Supplementary Fig. S2b, Supplementary Table S9). In these patients, the most common first-line regimens were XP plus T-mab (41.2%) and SP plus T-mab (11.8%). The median durations of first-line therapy, overall treatment, and the first- to second-line transition rates for all first-line regimens are presented in Supplementary Table S9.

## Discussion

This is the first large-scale administrative claims database study to describe adjuvant and post-recurrent treatment patterns in Japanese patients with GC in a real-world setting.

Our study showed that the use of fluoropyrimidine plus platinum or DS adjuvant therapy increased after these regimens were established in 2016 and 2019, respectively, while the use of S-1 adjuvant therapy decreased over time. The most commonly used combination regimen during September 2019–March 2022 was DS (20.0%), while two-thirds of patients (65%) still received S-1. According to 2019–2021 hospital data study from Japan, approximately 60% of Japanese patients with pStage II–III receiving adjuvant therapy after surgery had pStage II GC [21]. Although the current study could not examine treatment patterns by disease stage due to lack of available data, our results suggest that a substantial proportion of the patients with pStage III GC may still receive S-1 in the adjuvant setting.

Our study assessed the treatment patterns, including treatment duration as a surrogate indicator of clinical outcomes, among patients who experienced recurrence during or after adjuvant therapy. Survival data are not accurately captured in the MDV database; however, previous studies have indicated that real-world outcomes (e.g., time-to-treatment discontinuation or time-to-next treatment) correlate with survival outcomes [22–24]. The median durations of first-line and overall treatment in our study (4.9 and 9.2 months, respectively) were similar to those previously reported by Komatsu and colleagues in patients with advanced GC (5.8 and 10.2 months, respectively) [17]. Of note, however, the previous study only included patients who had received first-line fluoropyrimidine- or platinum-based regimens [17].

In our study, first-line and overall treatment durations and first- to second-line transition rates were similar in patients with early and late recurrence. This finding differs from those of the Shitara et al. study, in which the prognosis was worse among patients with early versus those with late recurrence [10]. Considering that this difference could be affected by the new agent, ramucirumab (added to the JGCA guidelines in October 2015 [11]), we evaluated the durations of first-line therapy and overall survival. In patients who started first-line therapy before October 2015, the median durations of first-line therapy and overall treatment were numerically shorter in the early-recurrent cohort than in the late-recurrent cohort, and similar to the study results of Shitara et al. [10]. Conversely, in patients who started first-line therapy after October 2015, the median durations of first-line therapy and overall treatment were similar between the early- and late-recurrent cohorts. However, the difference between our study findings and those of Shitara et al. [10] may also be due to their small population size (N = 52) and differences in study designs.

The optimal first-line treatment strategy for patients with early recurrence (≤ 6 months) after adjuvant therapy has not been established. As mentioned above, the JGCA guidelines recommend avoiding drugs that have already been used in the adjuvant setting in patients with recurrence within 6 months of adjuvant therapy [2, 11]. In the XParTS-I study, patients with early recurrence (≤ 6 months) after S-1–containing adjuvant therapy who received first-line XP therapy had a median progression-free survival (PFS) and overall survival (OS) of 4.4 and 13.7 months, respectively, indicating that switching to a different fluoropyrimidine was associated with a better clinical outcome in some patients [25]. However, there is no clear evidence to indicate whether another fluoropyrimidine, capecitabine, or a non-fluoropyrimidine second-line agent should be used in patients with recurrence who have previously received adjuvant S-1 therapy.

In our study, the most commonly used first-line regimens in the early-recurrent cohort of the T-mab/T-DXd [ −] group were paclitaxel or nab-paclitaxel plus ramucirumab (44.1%) and CapeOX (17.0%). This was in line with the JGCA guideline recommendations [11]. Interestingly, the median duration of first-line treatment was similar with CapeOX and the combination regimens of paclitaxel or nab-paclitaxel plus ramucirumab in the early-recurrent cohort; however, patients receiving first-line CapeOX had a higher first- to second-line transition rate and a longer overall treatment duration than those receiving paclitaxel or nab-paclitaxel plus ramucirumab. This may be because of differences in patient baseline characteristics between first-line regimens, as a higher proportion of the patients receiving first-line paclitaxel or nab-paclitaxel plus ramucirumab had peritoneal metastasis or ascites at baseline (which may be associated with reduced gastrointestinal absorption of oral drugs) compared with patients who received first-line CapeOX. Another possibility is that selection of first-line CapeOX therapy allows for more non-fluoropyrimidine–based second- and third-line therapy options than first-line paclitaxel or nab-paclitaxel plus ramucirumab, thereby increasing the first- to second-line transition rate and the overall duration of treatment.

In patients with late recurrence (≥ 6 months) after adjuvant therapy, management strategies are considered to be the same as those used for untreated patients in most trials of first-line therapy for unresectable advanced GC. Therefore, the JGCA guidelines recommend first-line fluoropyrimidine plus platinum chemotherapy in human epidermal growth factor receptor 2 (HER2)-negative patients with late recurrence [2]. Consistent with these recommendations, in our study, over half (55.6%) of the late recurrent cohort in the T-mab/T-DXd [ −] group received first-line fluoropyrimidine plus platinum chemotherapy. Similarly, in the retrospective study by Komatsu and colleagues, SOX and SP were the most common first-line regimens (in 40.9% and 23.8% of patients, respectively) [17].

Among the T-mab/T-DXd [ +] group of the recurrent cohort, > 80% received T-mab as part of their first-line regimen. Across both the early- and late-recurrent cohorts, 87% of patients who were treated with first-line T-mab received this as part of a recommended first-line regimen for patients with HER2-positive GC [2]. This suggests that, in patients with HER2-positive disease, the adjuvant therapy regimen does not affect selection of the first-line regimen, irrespective of the time to recurrence.

The optimal treatment strategy for older patients with GC has not been established, as these patients are often under-represented in clinical trials. In the adjuvant cohort of this study, the proportion who received S-1 adjuvant therapy in older patients (≥ 75 years) was higher than in the younger age groups. This may be due, at least in part, to the better safety profile of S-1 compared with platinum- or taxane-containing combination regimens. Although a trend towards clinical benefit with CapeOX and DS was previously reported in older patients, the incidence of grade 3/4 adverse events (AEs) was higher in patients receiving combination regimens than in those receiving S-1 [3, 5, 7]. However, interpretation of adjuvant treatment patterns among older patients in the current study should be made with caution, as data for patients who did not receive adjuvant therapy were not collected. In contrast, in the recurrent cohort, first-line treatment patterns were similar across the three age groups for the early-recurrent cohort, whereas, in the late-recurrent cohort, S-1 use was higher and SOX use was slightly lower among older (≥ 75 years) versus younger patients. In addition, the median overall treatment duration was numerically shorter in older versus younger patients. In a study in older patients (≥ 70 years) with unresectable or recurrent GC, first-line SOX provided survival benefits over S-1 monotherapy (median OS 16.2 vs 13.0 months; hazard ratio [HR] 0.73; 60% CI 0.63–0.84; p = 0.0535 and median PFS 6.5 vs 3.5 months; HR 0.56; 95% CI 0.49–0.65; p = 0.0004) [26]. However, some AEs (e.g., neutropenia, thrombocytopenia, and peripheral neuropathy) were more common with SOX versus S-1 [26], which would have been more apparent in older patients. Taken together, these data indicate that S-1 therapy may still be preferable to other JGCA-recommended regimens from the perspective of safety in older patients with late-recurrent GC.

This study has several limitations. The generalizability of these results to the overall GC patient population in Japan may be limited as the MDV database consists mainly of data from acute care hospitals with the DPC system. In addition, the database contains limited clinical and disease-related information, including TNM staging and recurrence status, and is unable to track patients beyond each hospital; therefore, analyses of treatment patterns by disease stage were not possible, patients may have been lost to follow-up, and later lines of therapy may not be accurately recorded. Further, as diagnostic information was based solely on medical claims data, the regimens and treatment lines were defined using a prespecified analysis algorithm. Finally, the current study may not reflect the latest treatment patterns in patients with recurrent GC in Japan, as these may have changed since the approval of first-line nivolumab-based combinations for advanced GC in November 2021 [27].

## Conclusions

This study provides important insights regarding adjuvant and post-recurrent treatment patterns among Japanese patients with GC in real-world clinical practice. Shifts in adjuvant treatment patterns over time were observed, including a decrease in the proportion of patients receiving S-1 in the later time periods after the SOX, CapeOX, and DS regimens were established. In the post-recurrent setting, paclitaxel or nab-paclitaxel plus ramucirumab and CapeOX were common first-line therapy regimens in the T-mab/T-DXd [ −] group of the early-recurrent cohort, while fluoropyrimidine plus platinum combinations were commonly used in those with late recurrence, consistent with the JGCA guidelines. These findings may guide future research into optimizing treatment strategies in patients with GC.

### Supplementary Information

Below is the link to the electronic supplementary material.

Supplementary file1 (DOCX 994 KB)

## Data Availability

Bristol Myers Squibb’s policy on data sharing may be found at https://www.bms.com/researchers-and-partners/independent-research/data-sharing-request-process.html.
