# Supplementary material for: Adjuvant and post-recurrent treatment patterns in patients with resectable gastric cancer in Japan: a retrospective database cohort study
Source: Gastric Cancer. 2024 Apr 30;27(4):827–39. doi: 10.1007/s10120-024-01501-w (PMC11193688; doi:10.1007/s10120-024-01501-w)
Supplement: Supplementary file 1 — Supplementary file1 (DOCX 994 KB) [file 10120_2024_1501_MOESM1_ESM.docx]

**Adjuvant and post-recurrent treatment patterns in patients with resectable gastric cancer in Japan: a retrospective database cohort study**

*Gastric Cancer*

**Authors:** Takaki Yoshikawa^1^*, Yorifumi Kikko^2^*, Reina Makino^3^, Yuya Kimijima^2^, Eiji Nishiyama^3^, Yuko Matsuda^3^, Bruno Casaes Teixeira^4^, Mariella Tejada^5^, Robert Carroll^4^, Shuichi Hironaka^6,7^

**Affiliations:** ^1^National Cancer Center Hospital, Tokyo, Japan; ^2^Bristol-Myers Squibb K.K., Tokyo, Japan; ^3^Ono Pharmaceutical Co., Ltd, Osaka, Japan; ^4^Bristol Myers Squibb, Uxbridge, UK; ^5^Bristol Myers Squibb, Princeton, New Jersey, USA; ^6^Kyorin University Faculty of Medicine, Tokyo, Japan; ^7^Saitama Medical University International Medical Center, Saitama, Japan

**Corresponding author:** Shuichi Hironaka; Email: [shuichi-hironaka@ks.kyorin-u.ac.jp](mailto:shuichi-hironaka@ks.kyorin-u.ac.jp)

# Supplementary material

## Supplementary methods

### Assessment of adjuvant therapy patterns

Assessment of adjuvant therapy was conducted separately in three treatment periods, based on the adjuvant therapy start date: (1) May 1, 2008 to May 31, 2016; (2) June 1, 2016 to August 31, 2019; and (3) September 1, 2019 to March 31, 2022. These dates were selected based on capecitabine plus oxaliplatin (CapeOX) and tegafur/gimeracil/oteracil potassium (S-1) plus oxaliplatin (SOX) being recommended by the Japanese Gastric Cancer Association (JGCA) guidelines in June 2016 [29] and docetaxel plus S-1 (DS) being recommended in September 2019 [30].

### Index treatment regimens

Adjuvant therapy was limited to the index regimens: S-1, SOX, CapeOX, and DS. First-line therapy after recurrence was limited to the index regimens (i.e., S-1 plus cisplatin [SP], capecitabine plus cisplatin [XP], SOX, CapeOX, folinic acid plus 5-fluorouracil [5-FU] plus oxaliplatin [FOLFOX], SP plus trastuzumab [T-mab], XP plus T-mab, SOX plus T-mab, CapeOX plus T-mab, 5-FU plus cisplatin, 5-FU plus folinic acid, 5-FU plus folinic acid plus paclitaxel, S-1, DS, 5-FU plus cisplatin plus trastuzumab, and FOLFOX plus T-mab).

## Supplementary Table S1

Insurance claims codes for diagnoses and medical procedures used in the database study

| ICD-10 diagnosis code | Description |
| --- | --- |
| C16 | Malignant neoplasm of stomach |
| C16.0 | Malignant neoplasm: Cardia |
| C16.1 | Malignant neoplasm: Fundus of stomach |
| C16.2 | Malignant neoplasm: Body of stomach |
| C16.3 | Malignant neoplasm: Pyloric antrum |
| C16.4 | Malignant neoplasm: Pylorus |
| C16.5 | Malignant neoplasm: Lesser curvature of stomach, unspecified |
| C16.6 | Malignant neoplasm: Greater curvature of stomach, unspecified |
| C16.8 | Malignant neoplasm: Overlapping lesion of stomach |
| C16.9 | Malignant neoplasm: Stomach, unspecified |
| Surgical procedure receipt code | Description |
| 150168110 | Total gastric resection (malignant tumor surgery) [K6572] |
| 150168010 | Gastric resection (malignant tumor surgery) [K6552] |
| 150323510 | Laparoscopic gastric local site resection (malignant tumor surgery) [K655-22] |
| 150323710 | Laparoscopic total gastric resection (malignant tumor surgery) [K657-22] |
| 150337310 | Proximal gastrectomy (malignant tumor resection) [K655-42] |
| 150407110 | Laparoscopic total gastric resection (malignant tumor surgery) (with supportive device for endoscopic surgery) [K657-22] |
| 150377910 | Laparoscopic proximal gastrectomy (malignant tumor resection) [K655-52] |
| 150406910 | Laparoscopic gastric partial resection (with malignant tumor resection/surgery support instrument) [K655-52] |
| 150406710 | Laparoscopic gastric local site resection (surgery for malignant tumor) (with supportive device for endoscopic surgery) [K655-22] |

## Supplementary Table S2

Insurance claims codes for Japanese Gastric Cancer Association guideline-recommended antitumor agents

| Receipt code | Generic name | Description |
| --- | --- | --- |
| 620001919 | Cisplatin | IA-call 100 mg for IA injection |
| 620002591 |  | IA-call 50 mg for IA injection |
| 620004129 |  | Cisplatin injection 10 mg/20 mL (NICHIIKO) |
| 620004130 |  | Cisplatin injection 25 mg/50 mL (NICHIIKO) |
| 620004131 |  | Cisplatin injection 50 mg/100 mL (NICHIIKO) |
| 620006298 |  | Briplatin injection 10 mg/20 mL |
| 620006299 |  | Briplatin injection 25 mg/50 mL |
| 620006300 |  | Briplatin injection 50 mg/100 mL |
| 620008946 |  | Randa injection 10 mg/20 mL |
| 620008947 |  | Randa injection 25 mg/50 mL |
| 620008948 |  | Randa injection 50 mg/100 mL |
| 620009545 |  | Cisplatin IV infusion 10 mg/20 mL (MYLAN) |
| 620009546 |  | Cisplatin IV infusion 25 mg/50 mL (MYLAN) |
| 620009547 |  | Cisplatin IV infusion 50 mg/100 mL (MYLAN) |
| 620923202 |  | Cisplatin IV infusion 10 mg/20 mL (PFIZER) |
| 620923301 |  | Cisplatin IV infusion 10 mg/20 mL (MARUKO) |
| 620923602 |  | Cisplatin IV infusion 25 mg/50 mL (PFIZER) |
| 620923701 |  | Cisplatin IV infusion 25 mg/50 mL (MARUKO) |
| 620924002 |  | Cisplatin IV infusion 50 mg/100 mL (PFIZER) |
| 620924101 |  | Cisplatin IV infusion 50 mg/100 mL (MARUKO) |
| 622760800 |  | Cisplatin IV infusion 10 mg/20 mL |
| 622760900 |  | Cisplatin IV infusion 25 mg/50 mL |
| 622761000 |  | Cisplatin IV infusion 50 mg/100 mL |
| 640406088 |  | Cisplatin injection 10 mg/20 mL (MARUKO) |
| 640406089 |  | Cisplatin injection 25 mg/50 mL (MARUKO) |
| 640406090 |  | Cisplatin injection 50 mg/100 mL (MARUKO) |
| 644210052 |  | Briplatin injection 50 mg/100 mL |
| 644210053 |  | Briplatin injection 25 mg/50 mL |
| 644210054 |  | Briplatin injection 10 mg/20 mL |
| 644210055 |  | Randa injection 50 mg/100 mL |
| 644210056 |  | Randa injection 25 mg/50 mL |
| 644210057 |  | Randa injection 10 mg/20 mL |
| 644290002 |  | Platosin injection 10 mg/20 mL |
| 644290003 |  | Platosin injection 25 mg/50 mL |
| 644290004 |  | Platosin injection 50 mg/100 mL |
| 620002490 | Oxaliplatin | Elplat for injection 100 mg |
| 620008572 |  | Elplat for injection 50 mg |
| 621932201 |  | Elplat IV infusion 50 mg/10 mL |
| 621932301 |  | Elplat IV infusion 100 mg/20 mL |
| 622189401 |  | Elplat IV infusion 200 mg/40 mL |
| 622371101 |  | Oxaliplatin IV infusion 50 mg/10 mL (DSEP) |
| 622371201 |  | Oxaliplatin IV infusion 100 mg/20 mL (DSEP) |
| 622371801 |  | Oxaliplatin IV infusion 50 mg/10 mL (TOWA) |
| 622371901 |  | Oxaliplatin IV infusion 100 mg/20 mL (TOWA) |
| 622373201 |  | Oxaliplatin IV infusion 50 mg/10 mL (FFP) |
| 622373301 |  | Oxaliplatin IV infusion 100 mg/20 mL (FFP) |
| 622374801 |  | Oxaliplatin IV infusion 50 mg/10 mL (HOSPIRA) |
| 622374901 |  | Oxaliplatin IV infusion 100 mg/20 mL (HOSPIRA) |
| 622381301 |  | Oxaliplatin IV infusion 50 mg/10 mL (PFIZER) |
| 622381401 |  | Oxaliplatin IV infusion 100 mg/20 mL (PFIZER) |
| 622383201 |  | Oxaliplatin IV infusion 50 mg/10 mL (SANDOZ) |
| 622383301 |  | Oxaliplatin IV infusion 100 mg/20 mL (SANDOZ) |
| 622385701 |  | Oxaliplatin IV infusion 50 mg/10 mL (NK) |
| 622385801 |  | Oxaliplatin IV infusion 100 mg/20 mL (NK) |
| 622388601 |  | Oxaliplatin IV infusion 50 mg/10 mL (CHEMIPHAR) |
| 622388701 |  | Oxaliplatin IV infusion 100 mg/20 mL (CHEMIPHAR) |
| 622389801 |  | Oxaliplatin IV infusion 50 mg/10 mL (SAWAI) |
| 622389901 |  | Oxaliplatin IV infusion 100 mg/20 mL (SAWAI) |
| 622392001 |  | Oxaliplatin IV infusion 50 mg/10 mL (NIPRO) |
| 622392101 |  | Oxaliplatin IV infusion 100 mg/20 mL (NIPRO) |
| 622393201 |  | Oxaliplatin IV infusion 50 mg/10 mL (NICHIIKO) |
| 622393301 |  | Oxaliplatin IV infusion 100 mg/20 mL (NICHIIKO) |
| 622394701 |  | Oxaliplatin IV infusion 50 mg/10 mL (TEVA) |
| 622394801 |  | Oxaliplatin IV infusion 100 mg/20 mL (TEVA) |
| 622411901 |  | Oxaliplatin IV infusion 200 mg/40 mL (TOWA) |
| 622417801 |  | Oxaliplatin IV infusion 200 mg/40 mL (PFIZER) |
| 622426801 |  | Oxaliplatin IV infusion 200 mg/40 mL (DSEP) |
| 622428001 |  | Oxaliplatin IV infusion 200 mg/40 mL (CHEMIPHAR) |
| 622431101 |  | Oxaliplatin IV infusion 200 mg/40 mL (SAWAI) |
| 622432401 |  | Oxaliplatin IV infusion 200 mg/40 mL (TEVA) |
| 622434901 |  | Oxaliplatin IV infusion 200 mg/40 mL (NK) |
| 622437001 |  | Oxaliplatin IV infusion 200 mg/40 mL (NICHIIKO) |
| 622437201 |  | Oxaliplatin IV infusion 50 mg/10 mL (KCC) |
| 622437301 |  | Oxaliplatin IV infusion 100 mg/20 mL (KCC) |
| 622437401 |  | Oxaliplatin IV infusion 200 mg/40 mL (KCC) |
| 622439101 |  | Oxaliplatin IV infusion 200 mg/40 mL (NIPRO) |
| 622460601 |  | Oxaliplatin IV infusion 200 mg/40 mL (HOSPIRA) |
| 622461701 |  | Oxaliplatin IV infusion 200 mg/40 mL (SANDOZ) |
| 622476900 |  | Oxaliplatin 100 mg/20 mL injection |
| 622617800 |  | Oxaliplatin 50 mg/10 mL injection |
| 622617900 |  | Oxaliplatin 200 mg/40 mL injection |
| 610470009 | Capecitabine | Xeloda tablets 300 mg |
| 622656401 |  | Capecitabine tablets 300 mg (SAWAI) |
| 622674301 |  | Capecitabine tablets 300 mg (NICHIIKO) |
| 622677701 |  | Capecitabine tablets 300 mg (TOWA) |
| 622679001 |  | Capecitabine tablets 300 mg (YAKULT) |
| 622695801 |  | Capecitabine tablets 300 mg (NK) |
| 622700101 |  | Capecitabine tablets 300 mg (JG) |
| 620005087 | Tegafur | Futraful enteric-coated granules 50% |
| 620004748 |  | Futraful injection 400 mg/10 mL |
| 620004566 |  | Futraful capsules 200 mg |
| 620004820 |  | Futraful suppositories 750 mg |
| 621930101 | Tegafur/uracil | UFT E combination granules 200 mg (equivalent tegafur) |
| 620003181 |  | UFT capsules (equivalent tegafur) |
| 620915001 |  | UFT combination capsules 100 mg (equivalent tegafur) |
| 621929901 |  | UFT E combination granules 100 mg (equivalent tegafur) |
| 620004602 |  | UFT E granules 20% (equivalent tegafur) |
| 621930001 |  | UFT E combination granules 150 mg (equivalent tegafur) |
| 610421353 | S-1 | TS-1 capsules 20 mg (equivalent tegafur) |
| 610421354 |  | TS-1 capsules 25 mg (equivalent tegafur) |
| 620009353 |  | TS-1 combination granules 20 mg (equivalent tegafur) |
| 620009354 |  | TS-1 combination granules 25 mg (equivalent tegafur) |
| 620915501 |  | TS-1 combination capsules 20 mg (equivalent tegafur) |
| 620915601 |  | TS-1 combination capsules 25 mg (equivalent tegafur) |
| 622243001 |  | TS-1 combination OD tablets 20 mg (equivalent tegafur) |
| 622243101 |  | TS-1 combination OD tablets 25 mg (equivalent tegafur) |
| 622254901 |  | NKS-1 combination capsules 20 mg (equivalent tegafur) |
| 622255001 |  | NKS-1 combination capsules 25 mg (equivalent tegafur) |
| 622256001 |  | Esueewan combination capsules 20 mg (equivalent tegafur) |
| 622256101 |  | Esueewan combination capsules 25 mg (equivalent tegafur) |
| 622275701 |  | Temeral combination capsules 20 mg (equivalent tegafur) |
| 622275801 |  | Temeral combination capsules 25 mg (equivalent tegafur) |
| 622285701 |  | S-1Meiji combination capsules 20 mg (equivalent tegafur) |
| 622294601 |  | S-1 NP combination capsules 20 mg (equivalent tegafur) |
| 622294701 |  | S-1 NP combination capsules 25 mg (equivalent tegafur) |
| 622397101 |  | EES-1 combination tablets 20 mg (equivalent tegafur) |
| 622397201 |  | EES-1 combination tablets 25 mg (equivalent tegafur) |
| 622397301 |  | S-1KK combination tablets 20 mg (equivalent tegafur) |
| 622397401 |  | S-1KK combination tablets 25 mg (equivalent tegafur) |
| 622430801 |  | Esueewan combination granules 20 mg (equivalent tegafur) |
| 622430901 |  | Esueewan combination granules 25 mg (equivalent tegafur) |
| 622434701 |  | NKS-1 combination granules 20 mg (equivalent tegafur) |
| 622434801 |  | NKS-1 combination granules 25 mg (equivalent tegafur) |
| 622487301 |  | NKS-1 combination OD tablets 20 mg (equivalent tegafur) |
| 622487401 |  | NKS-1 combination OD tablets 25 mg (equivalent tegafur) |
| 622497901 |  | Esueewan combination OD tablets 20 mg (equivalent tegafur) |
| 622498001 |  | Esueewan combination OD tablets 25 mg (equivalent tegafur) |
| 622537501 |  | S-1TAIHO combination OD tablets 20 mg (equivalent tegafur) |
| 622537601 |  | S-1TAIHO combination OD tablets 25 mg (equivalent tegafur) |
| 622285801 |  | S-1Meiji combination capsules 25 mg (equivalent tegafur) |
| 622047901 | 5-Fluorouracil | 5-FU injection 1000 mg |
| 622229101 |  | 5-FU injection 250 mg |
| 622412501 |  | Fluorouracil injection 250 mg (TOWA) |
| 622412601 |  | Fluorouracil injection 1000 mg (TOWA) |
| 640463105 |  | 5-FU injection 250 mg KYOWA |
| 622336001 | Trifluridine/tipiracil | Lonsurf combination tablets 15 mg (equivalent trifluridine) |
| 622336101 |  | Lonsurf combination tablets 20 mg (equivalent trifluridine) |
| 620003751 | Paclitaxel | Taxol injection 30 mg/5 mL |
| 620003752 |  | Taxol injection 100 mg/16.7 mL |
| 620004170 |  | Paclitaxel injection 30 mg/5 mL (NK) |
| 620004171 |  | Paclitaxel injection 100 mg/16.7 mL (NK) |
| 620005688 |  | Paclitaxel injection 30 mg/5 mL (SAWAI) |
| 620005689 |  | Paclitaxel injection 100 mg/16.7 mL (SAWAI) |
| 620005690 |  | Paclitaxel injection 150 mg/25 mL (SAWAI) |
| 622009101 |  | Paclitaxel injection 30 mg/5 mL (MYLAN) |
| 622009102 |  | Paclitaxel injection 30 mg/5 mL (PFIZER) |
| 622009201 |  | Paclitaxel injection 100 mg/16.7 mL (MYLAN) |
| 622009202 |  | Paclitaxel injection 100 mg/16.7 mL (PFIZER) |
| 622082001 |  | Paclitaxel IV infusion 30 mg/5 mL (SANDOZ) |
| 622082101 |  | Paclitaxel IV infusion 100 mg/16.7 mL (SANDOZ) |
| 622259101 |  | Paclitaxel injection 30 mg/5 mL (NP) |
| 622259201 |  | Paclitaxel injection 100 mg 16.7 mL (NP) |
| 622375001 |  | Paclitaxel IV infusion 30 mg/5 mL (HOSPIRA) |
| 622375101 |  | Paclitaxel IV infusion 100 mg/16.7 mL (HOSPIRA) |
| 622760500 |  | Paclitaxel IV infusion 30 mg/5 mL |
| 622760600 |  | Paclitaxel IV infusion 100 mg/16.7 mL |
| 622760700 |  | Paclitaxel IV infusion 150 mg/25 mL |
| 621970101 | nab-Paclitaxel | Abraxane for IV infusion 100 mg |
| 620007257 | Irinotecan | Campto IV infusion 40 mg/2 mL |
| 620007258 |  | Campto IV infusion 100 mg/5 mL |
| 620009515 |  | Irinotecan hydrochloride IV infusion 40 mg/2 mL (NK) |
| 620009516 |  | Irinotecan hydrochloride IV infusion 40 mg/2 mL (SAWAI) |
| 620009517 |  | Irinotecan hydrochloride IV infusion 40 mg/2 mL (SANDOZ) |
| 620009518 |  | Irinotecan hydrochloride IV infusion 40 mg/2 mL (TAIHO) |
| 620009519 |  | Irinotecan hydrochloride IV infusion 100 mg/5 mL (NK) |
| 620009520 |  | Irinotecan hydrochloride IV infusion 100 mg/5 mL (SAWAI) |
| 620009521 |  | Irinotecan hydrochloride IV infusion 100 mg/5 mL (SANDOZ) |
| 620009522 |  | Irinotecan hydrochloride IV infusion 100 mg/5 mL (TAIHO) |
| 620919501 |  | Topotecin IV infusion 40 mg/2 mL |
| 620919701 |  | Topotecin IV infusion 100 mg/5 mL |
| 621900302 |  | Irinotecan hydrochloride IV infusion 40 mg/2 mL (SUN) |
| 621900402 |  | Irinotecan hydrochloride IV infusion 100 mg/5 mL (SUN) |
| 622019401 |  | Irinotecan hydrochloride IV infusion 40 mg/2 mL (HOSPIRA) |
| 622019501 |  | Irinotecan hydrochloride IV infusion 100 mg/5 mL (HOSPIRA) |
| 622059701 |  | Irinotecan hydrochloride IV infusion 40 mg/2 mL (TAIYO) |
| 622059801 |  | Irinotecan hydrochloride IV infusion 100 mg/5 mL (TAIYO) |
| 622091101 |  | Irinotecan hydrochloride IV infusion 40 mg/2 mL (ASKA) |
| 622091201 |  | Irinotecan hydrochloride IV infusion 100 mg/5 mL (ASKA) |
| 622230201 |  | Irinotecan hydrochloride IV infusion 40 mg/2 mL (NICHIIKO) |
| 622230301 |  | Irinotecan hydrochloride IV infusion 100 mg/5 mL (NICHIIKO) |
| 622236901 |  | Irinotecan hydrochloride IV infusion 40 mg/2 mL (TOWA) |
| 622237001 |  | Irinotecan hydrochloride IV infusion 100 mg/5 mL (TOWA) |
| 622470401 |  | Irinotecan hydrochloride IV infusion 40 mg/2 mL (HENGRUI) |
| 622470501 |  | Irinotecan hydrochloride IV infusion 100 mg/5 mL (HENGRUI) |
| 622258901 |  | Irinotecan hydrochloride IV infusion 40 mg/2 mL (NP) |
| 622259001 |  | Irinotecan hydrochloride IV infusion 100 mg/5 mL (NP) |
| 644290006 |  | Campto injection 40 mg/2 mL |
| 644290007 |  | Campto injection 100 mg/5 mL |
| 644290008 |  | Topotecin injection 40 mg/2 mL |
| 644290009 |  | Topotecin injection 100 mg/5 mL |
| 620919801 | Docetaxel | Taxotere for IV infusion 20 mg/0.5 mL (w/solution) |
| 620919901 |  | Taxotere for IV infusion 80 mg/2 mL (w/solution) |
| 622068501 |  | Onetaxotere IV infusion 20 mg/1mL |
| 622068601 |  | Onetaxotere IV infusion 80 mg/4mL |
| 622215301 |  | Docetaxel IV infusion 20 mg/2 mL (SANDOZ) |
| 622215401 |  | Docetaxel IV infusion 80 mg/8mL (SANDOZ) |
| 622231801 |  | Docetaxel for IV infusion 20 mg/0.5 mL(w/solution) (ASKA) |
| 622231901 |  | Docetaxel for IV infusion 80 mg/2 mL(w/solution) (ASKA) |
| 622272001 |  | Docetaxel IV infusion 20 mg/1mL (TOWA) |
| 622272101 |  | Docetaxel IV infusion 80 mg/4mL (TOWA) |
| 622283101 |  | Docetaxel IV infusion 20 mg/1mL (TEVA) |
| 622283201 |  | Docetaxel IV infusion 80 mg/4mL (TEVA) |
| 622285201 |  | Docetaxel IV infusion 20 mg/2 mL (HOSPIRA) |
| 622285301 |  | Docetaxel IV infusion 80 mg/8mL (HOSPIRA) |
| 622285401 |  | Docetaxel IV infusion 120 mg/12 mL (HOSPIRA) |
| 622290401 |  | Docetaxel IV infusion 20 mg/1mL (HK) |
| 622290501 |  | Docetaxel IV infusion 80 mg/4mL (HK) |
| 622294901 |  | Docetaxel IV infusion 20 mg/1mL (CHEMIPHAR) |
| 622295001 |  | Docetaxel IV infusion 80 mg/4mL (CHEMIPHAR) |
| 622295501 |  | Docetaxel for IV infusion 20 mg/0.5 mL (w/solution) (SAWAI) |
| 622295601 |  | Docetaxel for IV infusion 80 mg/2 mL (w/solution) (SAWAI) |
| 622354801 |  | Docetaxel IV infusion 20 mg/1mL (NK) |
| 622354901 |  | Docetaxel IV infusion 80 mg/4mL (NK) |
| 622356401 |  | Docetaxel IV infusion 20 mg/1mL (SAWAI) |
| 622356501 |  | Docetaxel IV infusion 80 mg/4mL (SAWAI) |
| 622408501 |  | Docetaxel IV infusion 20 mg/1mL (YAKULT) |
| 622408601 |  | Docetaxel IV infusion 80 mg/4mL (YAKULT) |
| 622417601 |  | Docetaxel IV infusion 20 mg/1mL (PFIZER) |
| 622417701 |  | Docetaxel IV infusion 80 mg/4mL (PFIZER) |
| 622429301 |  | Docetaxel IV infusion 20 mg/1mL (EE) |
| 622429401 |  | Docetaxel IV infusion 80 mg/4mL (EE) |
| 622435002 |  | Docetaxel IV infusion 20 mg/1mL (NIPRO) |
| 622435102 |  | Docetaxel IV infusion 80 mg/4mL (NIPRO) |
| 640411025 |  | Taxotere injection 20 mg/0.5 mL (w/solution) |
| 640411026 |  | Taxotere injection 80 mg/2 mL (w/solution) |
| 620001938 | Trastuzumab | Herceptin for injection 60 mg (w/solution, dilution) |
| 622069801 |  | Herceptin for injection 60 mg (w/solution) |
| 622069901 |  | Herceptin for injection 150 mg (w/solution) |
| 622630701 |  | Trastuzumab BS for IV infusion 60 mg (NK) |
| 622630801 |  | Trastuzumab BS for IV infusion 150 mg (NK) |
| 622659701 |  | Trastuzumab BS for IV infusion 60 mg (DAIICHI-SANKYO) (w/solution) |
| 622659801 |  | Trastuzumab BS for IV infusion 150 mg (DAIICHI-SANKYO) (w/solution) |
| 622679201 |  | Trastuzumab BS for IV infusion 60 mg (PFIZER) |
| 622679301 |  | Trastuzumab BS for IV infusion 150 mg (PFIZER) |
| 640451013 |  | Herceptin for injection 150 mg (w/solution, dilution) |
| 622628901 |  | Trastuzumab BS for IV infusion 60 mg (CTH) |
| 622629001 |  | Trastuzumab BS for IV infusion 150 mg (CTH) |
| 629907101 | Trastuzumab deruxtecan | Enhertu for IV infusion 100 mg |
| 622417901 | Ramucirumab | Cyramza IV infusion 100 mg/10 mL |
| 622418001 |  | Cyramza IV infusion 500 mg/50 mL |
| 622364801 | Nivolumab | Opdivo IV infusion 20 mg/2 mL |
| 622364901 |  | Opdivo IV infusion 100 mg/10 mL |
| 622662201 |  | Opdivo IV infusion 240 mg/24mL |
| 629911501 |  | Opdivo IV infusion 120 mg/12 mL |
| 622515701 | Pembrolizumab | Keytruda IV infusion 20 mg/0.8mL |
| 622515801 |  | Keytruda IV infusion 100 mg/4mL |
| 620005715 | Levofolinate calcium | Revo folinate IV infusion 25 mg (OHARA) |
| 620005716 |  | Revo folinate IV infusion 25 mg (HK) |
| 620005717 |  | Revo folinate IV infusion 25 mg (NK) |
| 620005718 |  | Revo folinate IV infusion 25 mg (NP) |
| 620005719 |  | Revo folinate IV infusion 25 mg (F) |
| 620005720 |  | Revo folinate IV infusion 25 mg (SAWAI) |
| 620005721 |  | Revo folinate IV infusion 25 mg (TAIYO) |
| 620005722 |  | Revo folinate IV infusion 25 mg (TOWA) |
| 620005723 |  | Revo folinate IV infusion 25 mg (NICHIIKO) |
| 620005724 |  | Revo folinate IV infusion 25 mg (BT) |
| 620005725 |  | Revo folinate IV infusion 25 mg (YAKULT) |
| 620005726 |  | Revo folinate IV infusion 50 mg (NICHIIKO) |
| 620005727 |  | Revo folinate IV infusion 100 mg (OHARA) |
| 620005728 |  | Revo folinate IV infusion 100 mg (NK) |
| 620005729 |  | Revo folinate IV infusion 100 mg (TOWA) |
| 620005730 |  | Revo folinate IV infusion 100 mg (YAKULT) |
| 620005879 |  | Revo folinate IV infusion 100 mg (HK) |
| 620005880 |  | Revo folinate IV infusion 100 mg (NP) |
| 620005881 |  | Revo folinate IV infusion 100 mg (SAWAI) |
| 620008234 |  | Revo folinate IV infusion 100 mg (F) |
| 620008543 |  | Revo folinate IV infusion 100 mg (NICHIIKO) |
| 620009589 |  | Revo folinate IV infusion 100 mg (TAIYO) |
| 620009590 |  | Revo folinate IV infusion 100 mg (BT) |
| 622693500 |  | Revo folinate calcium for injection 25 mg |
| 622758000 |  | Revo folinate calcium for injection 100 mg |
| 622758100 |  | Revo folinate calcium for injection 50 mg |

*5-FU* 5-fluorouracil, *BCG* Bacillus Calmette–Guérin, *IA* intra-arterial, *ICD-10* International Classification of Diseases 10^th^ revision, *IV* intravenous, *S-1* tegafur/gimeracil/oteracil potassium, *OD* once daily, *SC* subcutaneous, *w/* with

## Supplementary Table S3

Patient demographics and hospital information in T-mab/T-DXd [+] group in the recurrent cohort

|  | Early recurrence in ≤180 days (n = 89) | Late recurrence in >180 days (n = 34) |
| --- | --- | --- |
| Male, n (%) | 69 (77.5) | 28 (82.4) |
| Age,^a^ years, median (range) | 70 (46–83) | 69.5 (44–83) |
| Age category, n (%) |  |  |
| <65 years | 16 (18.0) | 8 (23.5) |
| 65–74 years | 50 (56.2) | 19 (55.9) |
| ≥75 years | 23 (25.8) | 7 (20.6) |
| Comorbidities,^b^ n (%) |  |  |
| Hypertension | 24 (27.0) | 4 (11.8) |
| Diabetes | 17 (19.1) | 4 (11.8) |
| Liver disease | 15 (16.9) | 4 (11.8) |
| Ischemic heart disease | 1 (1.1) | 4 (11.8) |
| Thrombosis | 6 (6.7) | 0 |
| Kidney disease | 5 (5.6) | 1 (2.9) |
| Hemorrhoids | 1 (1.1) | 0 |
| Neuropathy | 10 (11.2) | 4 (11.8) |
| Edema | 5 (5.6) | 0 |
| ADL index score, n (%) |  |  |
| Dependent | 2 (2.3) | 2 (5.9) |
| Independent | 65 (73.0) | 22 (64.7) |
| Missing/incomplete | 22 (24.7) | 10 (29.4) |
| Metastasis site, n (%) |  |  |
| Peritoneal (or ascites) | 8 (9.0) | 5 (14.7) |
| Lymph node | 12 (13.5) | 8 (23.5) |
| Liver | 17 (19.1) | 7 (20.6) |
| Lung | 5 (5.6) | 2 (5.9) |
| Bone | 2 (2.3) | 1 (2.9) |
| Brain | 0 | 6 (17.6) |
| Designated cancer hospital,^b^ n (%) | 70 (78.7) | 29 (85.3) |
| Department,^c^ n (%) |  |  |
| Internal medicine^d^ | 12 (13.5) | 4 (11.8) |
| Surgery^e^ | 71 (79.8) | 29 (85.3) |
| Other or unknown | 7 (7.9) | 3 (8.8) |
| Number of beds in hospital, n (%) |  |  |
| <200 | 8 (9.0) | 3 (8.8) |
| ≥200 to <500 | 41 (46.1) | 15 (44.1) |
| ≥500 | 40 (44.9) | 16 (47.1) |

*ADL* Activities of Daily Living, *T-mab/T-DXd [+]* with history of trastuzumab or trastuzumab deruxtecan treatment

^a^At start of adjuvant first-line therapy

^b^In the 180 days prior to the start of adjuvant first-line therapy

^c^Patients may have multiple medical departments recorded

^d^Includes internal medicine, rheumatology and collagen disease internal medicine, and gastroenterology internal medicine departments

^e^Includes breast and thyroid, cardiovascular, neurosurgery, pediatric, hepato-biliary-pancreatic, cosmetic, dental-oral, general, respiratory, gastroenterological, and plastic surgery departments

## Supplementary Table S4

Post-recurrent first-line therapy regimen patterns in T-mab/T-DXd [−] group of the recurrent cohort in patients with peritoneal metastasis or ascites in the early and late recurrent cohorts

| First-line regimen, n/N (%)^a^ | Peritoneal metastasis or ascites (n = 378) | |
| --- | --- | --- |
|  | Early recurrence (n = 172) | Late recurrence (n = 206) |
| With fluoropyrimidines |  |  |
| CapeOX | 17/113 (15.0) | 29/83 (34.9) |
| SP | 5/57 (8.8) | 22/91 (24.2) |
| XP | 4/32 (12.5) | 4/13 (30.8) |
| SOX | 4/30 (13.3) | 52/156 (33.3) |
| S-1 | 2/20 (10.0) | 12/78 (15.4) |
| FOLFOX | 2/7 (28.6) | 14/19 (73.7) |
| DS | 0/4 | 3/6 (50.0) |
| FOL + 5-FU | 1/1 (100.0) | 4/5 (80.0) |
| FOL + 5-FU + cisplatin | 0/0 | 0/1 |
| FOL + 5-FU + PTX | 0/0 | 0/1 |
| Without fluoropyrimidines |  |  |
| PTX + RAM | 71/226 (31.4) | 26/64 (40.6) |
| nab-PTX + RAM | 24/67 (35.8) | 17/29 (58.6) |
| PTX | 28/61 (45.9) | 13/27 (48.1) |
| nab-PTX | 6/21 (28.6) | 6/9 (66.7) |
| RAM | 3/15 (20.0) | 2/5 (40.0) |
| DTX | 5/6 (83.3) | 2/3 (66.7) |
| IRI | 0/3 | 0/2 |
| IRI + RAM | 0/1 | 0/0 |
| PEM | 0/0 | 0/1 |

*5-FU* 5-fluorouracil, *CapeOX* capecitabine plus oxaliplatin, *DTX* docetaxel, *DS* S-1 plus docetaxel, *FOL* folinic acid, *FOLFOX* folinic acid, 5-fluorouracil, and oxaliplatin, *IRI* irinotecan, *nab-PTX* nab-paclitaxel, *PEM* pembrolizumab, *PTX* paclitaxel, *S-1* tegafur/gimeracil/oteracil potassium, *RAM* ramucirumab, *SOX* S-1 plus oxaliplatin, *SP* S-1 plus cisplatin, *T-mab/T-DXd [−]* with no history of trastuzumab or trastuzumab deruxtecan treatment, *XP* capecitabine plus cisplatin

^a^For each percentage value, the denominator (N) was the total number of patients in the T-mab/T-DXd [−] group of the recurrent cohort who received the first-line regimen

## Supplementary Table S5

Post-recurrent first-line treatment regimen patterns in T-mab/T-DXd [−] group of the recurrent cohort by definition of early versus late recurrence (first sensitivity analysis)

| Regimen, n (%) | Early recurrence | | Late recurrence | |
| --- | --- | --- | --- | --- |
|  | ≤180 days (n = 664) | ≤210 days (n = 710) | >180 days (n = 593) | >210 days (n = 547) |
| With fluoropyrimidines |  |  |  |  |
| CapeOX | 113 (17.0) | 120 (16.9) | 83 (14.0) | 76 (13.9) |
| SP | 57 (8.6) | 65 (9.2) | 91 (15.4) | 83 (15.2) |
| XP | 32 (4.8) | 33 (4.7) | 13 (2.2) | 12 (2.2) |
| SOX | 30 (4.5) | 34 (4.8) | 156 (26.3) | 152 (27.8) |
| S-1 | 20 (3.0) | 23 (3.2) | 78 (13.2) | 75 (13.7) |
| FOLFOX | 7 (1.1) | 9 (1.3) | 19 (3.2) | 17 (3.1) |
| DS | 4 (0.6) | 4 (0.6) | 6 (1.0) | 6 (1.1) |
| FOL + 5-FU | 1 (0.2) | 1 (0.1) | 5 (0.8) | 5 (0.9) |
| FOL + 5-FU + cisplatin | 0 | 1 (0.1) | 1 (0.2) | 0 |
| FOL + 5-FU + PTX | 0 | 0 | 1 (0.2) | 1 (0.2) |
| Without fluoropyrimidines |  |  |  |  |
| PTX + RAM | 226 (34.0) | 235 (33.1) | 64 (10.8) | 55 (10.1) |
| nab-PTX + RAM | 67 (10.1) | 72 (10.1) | 29 (4.9) | 24 (4.4) |
| PTX | 61 (9.2) | 65 (9.2) | 27 (4.6) | 23 (4.2) |
| nab-PTX | 21 (3.2) | 22 (3.1) | 9 (1.5) | 8 (1.5) |
| RAM | 15 (2.3) | 15 (2.1) | 5 (0.8) | 5 (0.9) |
| DTX | 6 (0.9) | 7 (1.0) | 3 (0.5) | 2 (0.4) |
| IRI | 3 (0.5) | 3 (0.4) | 2 (0.3) | 2 (0.4) |
| IRI + RAM | 1 (0.2) | 1 (0.1) | 0 | 0 |
| PEM | 0 | 0 | 1 (0.2) | 1 (0.2) |

*5-FU* 5-fluorouracil, *CapeOX* capecitabine plus oxaliplatin, *DTX* docetaxel, *DS* S-1 plus docetaxel, *FOL* folinic acid, *FOLFOX* folinic acid, 5-fluorouracil, and oxaliplatin, *IRI* irinotecan, *nab-PTX* nab-paclitaxel, *PEM* pembrolizumab, *PTX* paclitaxel, *RAM* ramucirumab, *S-1* tegafur/gimeracil/oteracil potassium, *SOX* S-1 plus oxaliplatin, *SP* S-1 plus cisplatin, *T-mab/T-DXd [−]* with no history of trastuzumab or trastuzumab deruxtecan treatment, *XP* capecitabine plus cisplatin

## Supplementary Table S6

Post-recurrent first-line therapy regimen patterns in T-mab/T-DXd [−] group of the recurrent cohort by definition of regimen end (second sensitivity analysis)

| Regimen, n (%) | Early recurrence | | Late recurrence | |
| --- | --- | --- | --- | --- |
|  | 120 days (n = 664) | 90 days (n = 689) | 120 days (n = 593) | 90 days (n = 596) |
| With fluoropyrimidines |  |  |  |  |
| CapeOX | 113 (17.0) | 108 (15.7) | 83 (14.0) | 85 (14.3) |
| SP | 57 (8.6) | 60 (8.7) | 91 (15.4) | 87 (14.6) |
| XP | 32 (4.8) | 31 (4.5) | 13 (2.2) | 14 (2.4) |
| SOX | 30 (4.5) | 33 (4.8) | 156 (26.3) | 153 (25.7) |
| S-1 | 20 (3.0) | 66 (9.6) | 78 (13.2) | 86 (14.4) |
| FOLFOX | 7 (1.1) | 5 (0.7) | 19 (3.2) | 20 (3.4) |
| DS | 4 (0.6) | 4 (0.6) | 6 (1.0) | 6 (1.0) |
| FOL + 5-FU | 1 (0.2) | 1 (0.2) | 5 (0.8) | 4 (0.7) |
| FOL + 5-FU + cisplatin | 0 | 0 | 1 (0.2) | 1 (0.2) |
| FOL + 5-FU + PTX | 0 | 0 | 1 (0.2) | 1 (0.2) |
| Without fluoropyrimidines |  |  |  |  |
| PTX + RAM | 226 (34.0) | 215 (31.2) | 64 (10.8) | 62 (10.4) |
| nab-PTX + RAM | 67 (10.1) | 63 (9.1) | 29 (4.9) | 30 (5.0) |
| PTX | 61 (9.2) | 61 (8.9) | 27 (4.6) | 27 (4.5) |
| nab-PTX | 21 (3.2) | 19 (2.8) | 9 (1.5) | 10 (1.7) |
| RAM | 15 (2.3) | 15 (2.2) | 5 (0.8) | 5 (0.8) |
| DTX | 6 (0.9) | 4 (0.6) | 3 (0.5) | 3 (0.5) |
| IRI | 3 (0.5) | 3 (0.4) | 2 (0.3) | 2 (0.3) |
| IRI + RAM | 1 (0.2) | 1 (0.2) | 0 | 0 |
| PEM | 0 | 0 | 1 (0.2) | 0 |

*5-FU* 5-fluorouracil, *CapeOX* capecitabine plus oxaliplatin, *DTX* docetaxel, *DS* S-1 plus docetaxel, *FOL* folinic acid, *FOLFOX* folinic acid, 5-fluorouracil, and oxaliplatin, *IRI* irinotecan, *nab-PTX* nab-paclitaxel, *PEM* pembrolizumab, *PTX* paclitaxel, *S-1* tegafur/gimeracil/oteracil potassium, *RAM* ramucirumab, *SOX* S-1 plus oxaliplatin, *SP* S-1 plus cisplatin, *T-mab/T-DXd [−]* with no history of trastuzumab or trastuzumab deruxtecan treatment, *XP* capecitabine plus cisplatin

## Supplementary Table S7

Post-recurrent first-line treatment patterns in the T-mab/T-DXd [–] group of the recurrent cohort in patients who started first-line therapy **(a)** before October 2015 and **(b)** after October 2015

**(a)**

|  | Early recurrence^a^ (n = 74) | | | | Late recurrence^b^ (n = 62) | | | |
| --- | --- | --- | --- | --- | --- | --- | --- | --- |
|  | Patients (%) | 1L treatment duration (months)^c^ | 1L→2L transition (%) | Overall treatment duration (months)^c^ | Patients (%) | 1L treatment duration (months)^c^ | 1L→2L transition (%) | Overall treatment duration (months)^c^ |
| Total |  | 3.5 (2.8–4.7) | 52.2 | 7.6 (4.7–10.2) |  | 4.9 (2.9–5.9) | 53.4 | 10.0 (5.1–12.3) |
| With fluoropyrimidines |  |  |  |  |  |  |  |  |
| CapeOX | 8 (10.8) | 5.4 (2.3–7.4) | 87.5 | 11.9 (6.2–19.4) | 1 (1.6) | 5.8 | 100.0 | 19.2 |
| SP | 16 (21.6) | 4.8 (2.3–9.6) | 81.3 | 10.2 (4.7–25.2) | 16 (25.8) | 5.2 (2.8–6.9) | 85.7 | 14.3 (5.1–NR) |
| XP | 8 (10.8) | 2.7 (0.5–6.6) | 50.0 | 4.8 (0.5–29.0) | 4 (6.5) | 6.9 (3.3–8.5) | 75.0 | 10.7 (3.3–22.8) |
| SOX | 3 (4.1) | 4.3 (3.5–6.2) | 66.7 | 12.8 (0.3–NR) | 10 (16.1) | 5.7 (1.0–9.5) | 50.0 | 10.5 (1.0–18.9) |
| S-1 | 2 (2.7) | 1.2 (0.5–1.9) | 50.0 | 7.1 (0.5–13.7) | 14 (22.6) | 2.2 (0.5–5.0) | 35.7 | 3.7 (0.5–10.2) |
| FOLFOX | 0 | – | – | – | 0 | – | – | – |
| DS | 2 (2.7) | 6.9 (6.2–7.5) | 50.0 | 7.6 (6.2–9.0) | 2 (3.2) | 5.7 (0.8–10.6) | 50.0 | 10.3 (10.0–10.6) |
| FOL + 5-FU | 0 | – | – | – | 0 | – | – | – |
| FOL + 5-FU + cisplatin | 0 | – | – | – | 0 | – | – | – |
| FOL + 5-FU + PTX | 0 | – | – | – | 0 | – | – | – |
| Without fluoropyrimidines |  |  |  |  |  |  |  |  |
| PTX + RAM | 5 (6.8) | 8.1 (0.5–NR) | 0.0 | 8.1 (0.5–NR) | 2 (3.2) | NR (2.8–NR) | 0.0 | NR (2.8–NR) |
| nab-PTX + RAM | 0 | – | – | – | 0 | – | – | – |
| PTX | 21 (28.4) | 3.3 (1.1–4.7) | 21.1 | 3.3 (1.1–7.3) | 9 (14.5) | 2.6 (0.6–8.1) | 37.5 | 2.6 (0.6–11.5) |
| nab-PTX | 4 (5.4) | 1.4 (0.1–2.8) | 25.0 | 1.4 (0.1–9.3) | 3 (4.8) | 5.8 (1.6–7.2) | 33.3 | 5.8 (1.6–13.9) |
| RAM | 0 | – | – | – | 0 | – | – | – |
| DTX | 3 (4.1) | 2.7 (1.6–4.2) | 66.7 | 10.7 (1.6–10.7) | 1 (1.6) | 34.4 | 0.0 | 34.4 |
| IRI | 2 (2.7) | 2.4 (1.4–3.4) | 50.0 | 18.9 (1.4–36.3) | 0 | – | – | – |
| IRI + RAM | 0 | – | – | – | 0 | – | – | – |
| PEM | 0 | – | – | – | 0 | – | – | – |

*1L* first line, *2L* second line, *5-FU* 5-fluorouracil, *CapeOX* capecitabine plus oxaliplatin, *DTX* docetaxel, *DS* S-1 plus docetaxel, *FOL* folinic acid, *FOLFOX* folinic acid plus 5-fluorouracil plus oxaliplatin, *IRI* irinotecan, *NA* not available, *nab-PTX* nab-paclitaxel, *NR* not reached, *PEM* pembrolizumab, *PTX* paclitaxel, *RAM* ramucirumab, *S-1* tegafur/gimeracil/oteracil potassium, *SOX* S-1 plus oxaliplatin, *SP* S-1 plus cisplatin, *T-mab/T-DXd [–]* with no history of trastuzumab or trastuzumab deruxtecan treatment, *XP* capecitabine plus cisplatin

^a^Post-recurrent first-line therapy started ≤180 days after end of adjuvant therapy

^b^Post-recurrent first-line therapy started >180 days after end of adjuvant therapy

^c^Data presented as median (95% confidence interval)

**(b)**

|  | Early recurrence^a^ (n = 590) | | | | Late recurrence^b^ (n = 531) | | | |
| --- | --- | --- | --- | --- | --- | --- | --- | --- |
|  | Patients (%) | 1L treatment duration (months)^c^ | 1L→2L transition (%) | Overall treatment duration (months)^c^ | Patients (%) | 1L treatment duration (months)^c^ | 1L→2L transition (%) | Overall treatment duration (months)^c^ |
| Total |  | 4.6 (4.2–4.9) | 56.4 | 9.3 (7.8–10.6) |  | 4.9 (4.5–5.2) | 55.4 | 9.5 (8.3–10.4) |
| With fluoropyrimidines |  |  |  |  |  |  |  |  |
| CapeOX | 105 (17.8) | 4.6 (3.7–5.1) | 68.4 | 11.9 (9.0–15.9) | 82 (15.4) | 4.9 (4.2–6.0) | 58.1 | 9.7 (7.2–11.5) |
| SP | 41 (6.9) | 3.9 (2.8–4.5) | 68.4 | 14.5 (6.5–18.2) | 75 (14.1) | 5.1 (4.2–6.1) | 62.9 | 9.7 (7.6–13.6) |
| XP | 24 (4.1) | 4.5 (2.6–9.7) | 76.5 | 12.9 (8.8–29.5) | 9 (1.7) | 3.3 (1.9–5.1) | 55.6 | 4.6 (1.9–14.3) |
| SOX | 27 (4.6) | 5.4 (2.8–5.8) | 64.0 | 7.3 (5.4–18.4) | 146 (27.5) | 5.1 (4.2–5.9) | 61.2 | 10.4 (8.0–14.0) |
| S-1 | 18 (3.1) | 5.0 (3.0–7.2) | 66.7 | 24.8 (4.9–32.9) | 64 (12.1) | 4.9 (4.2–6.0) | 38.9 | 9.5 (6.4–11.4) |
| FOLFOX | 7 (1.2) | 3.6 (1.0–7.3) | 57.1 | 7.3 (1.4–NR) | 19 (3.6) | 4.6 (1.1–6.0) | 46.7 | 13.4 (1.1–33.3) |
| DS | 2 (0.3) | 10.2 (7.9–12.5) | 100.0 | NR (18.5–NR) | 4 (0.8) | 5.6 (4.1–NR) | 33.3 | 8.2 (4.1–10.4) |
| FOL + 5-FU | 1 (0.2) | 1.2 | 100.0 | 3.1 | 5 (0.9) | 2.8 (0.1–15.1) | 60.0 | 5.0 (0.1–18.7) |
| FOL + 5-FU + cisplatin | 0 | – | – | – | 1 (0.2) | 1.1 | 100.0 | 42.3 |
| FOL + 5-FU + PTX | 0 | – | – | – | 1 (0.2) | 3.4 | 100.0 | NA |
| Without fluoropyrimidines |  |  |  |  |  |  |  |  |
| PTX + RAM | 221 (37.5) | 4.9 (4.6–5.6) | 53.3 | 8.8 (7.2–10.9) | 62 (11.7) | 5.6 (3.9–6.8) | 50.9 | 8.9 (5.9–12.3) |
| nab-PTX + RAM | 67 (11.4) | 4.9 (3.5–5.6) | 44.8 | 6.5 (5.0–12.0) | 29 (5.5) | 5.1 (3.7–8.1) | 52.2 | 8.9 (5.1–17.6) |
| PTX | 40 (6.8) | 3.5 (1.4–5.1) | 36.4 | 3.6 (1.4–11.1) | 18 (3.4) | 4.2 (1.4–6.5) | 58.8 | 6.5 (2.4–17.4) |
| nab-PTX | 17 (2.9) | 3.7 (2.1–7.0) | 35.7 | 4.7 (2.1–20.0) | 6 (1.1) | 2.3 (1.4–13.6) | 50.0 | 11.1 (1.4–NR) |
| RAM | 15 (2.5) | 4.0 (0.1–5.3) | 40.0 | 6.7 (0.1–10.6) | 5 (0.9) | 2.6 (0.1–3.3) | 25.0 | 3.3 (0.1–11.1) |
| DTX | 3 (0.5) | 5.6 (1.4–5.6) | 50.0 | 7.4 (1.4–7.4) | 2 (0.4) | 4.8 (4.7–4.9) | 50.0 | 5.7 (4.9–6.6) |
| IRI | 1 (0.2) | 8.8 | 100.0 | 13.7 | 2 (0.4) | 2.6 | 0.0 | NR (2.6–NR) |
| IRI + RAM | 1 (0.2) | 3.5 | 0.0 | 3.5 | 0 | – | – | – |
| PEM | 0 | – | – | – | 1 (0.2) | 0.1 | 0.0 | 0.1 |

*1L* first line, *2L* second line, *5-FU* 5-fluorouracil, *CapeOX* capecitabine plus oxaliplatin, *DTX* docetaxel, *DS* S-1 plus docetaxel, *FOL* folinic acid, *FOLFOX* folinic acid plus 5-fluorouracil plus oxaliplatin, *IRI* irinotecan, *NA* not available, *nab-PTX* nab-paclitaxel, *NR* not reached, *PEM* pembrolizumab, *PTX* paclitaxel, *RAM* ramucirumab, *S-1* tegafur/gimeracil/oteracil potassium, *SOX* S-1 plus oxaliplatin, *SP* S-1 plus cisplatin, *T-mab/T-DXd [–]* with no history of trastuzumab or trastuzumab deruxtecan treatment, *XP* capecitabine plus cisplatin

^a^Post-recurrent first-line therapy started ≤180 days after end of adjuvant therapy

^b^Post-recurrent first-line therapy started >180 days after end of adjuvant therapy

^c^Data presented as median (95% confidence interval)

## Supplementary Table S8

Post-recurrent first-line treatment patterns in the T-mab/T-DXd [–] group of the recurrent cohort in patients aged **(a)** <65 years, **(b)** 65–74 years, and **(c)** ≥75 years

**(a)**

|  | Early recurrence^a^ (n = 147) | | | | Late recurrence^b^ (n = 168) | | | |
| --- | --- | --- | --- | --- | --- | --- | --- | --- |
|  | Patients (%) | 1L treatment duration (months)^c^ | 1L→2L transition (%) | Overall treatment duration (months)^c^ | Patients (%) | 1L treatment duration (months)^c^ | 1L→2L transition (%) | Overall treatment duration (months)^c^ |
| Total |  | 4.7 (4.1–5.1) | 60.6 | 9.5 (6.4–10.6) |  | 5.0 (4.3–5.6) | 60.3 | 10.4 (8.9–13.2) |
| With fluoropyrimidines |  |  |  |  |  |  |  |  |
| CapeOX | 25 (17.0) | 4.7 (3.0–5.6) | 66.7 | 9.1 (5.6–15.0) | 26 (15.5) | 4.9 (3.6–7.2) | 54.2 | 10.3 (4.4–16.6) |
| SP | 17 (11.6) | 3.5 (2.3–8.2) | 88.2 | 16.4 (7.8–25.2) | 36 (21.4) | 6.4 (4.3–7.2) | 70.6 | 14.3 (7.6–42.4) |
| XP | 7 (4.8) | 4.5 (2.6–10.4) | 100.0 | 13.9 (7.0–38.0) | 4 (2.4) | 4.1 (2.6–10.4) | 75.0 | 12.4 (4.6–14.3) |
| SOX | 8 (5.4) | 4.9 (2.8–6.5) | 75.0 | 10.1 (3.4–NR) | 44 (26.2) | 4.4 (3.5–6.2) | 67.5 | 11.3 (7.5–15.6) |
| S-1 | 6 (4.1) | 6.9 (4.9–9.2) | 66.7 | 22.6 (4.9–NR) | 9 (5.4) | 6.0 (0.5–9.4) | 37.5 | 6.8 (0.5–31.2) |
| FOLFOX | 1 (0.7) | 5.2 | 100.0 | NR (NR–NR) | 7 (4.2) | 7.1 (0.1–11.8) | 50.0 | 13.4 (0.1–33.3) |
| DS | 1 (0.7) | 12.5 | 100.0 | 18.5 | 2 (1.2) | 4.6 (4.1–5.1) | 50.0 | 7.3 (4.1–10.4) |
| FOL + 5-FU | 0 | – | – | – | 2 (1.2) | 1.4 (0.1–2.8) | 50.0 | 2.5 (0.1–5.0) |
| FOL + 5-FU + cisplatin | 0 | – | – | – | 1 (0.6) | 1.1 | 100.0 | 42.3 |
| FOL + 5-FU + PTX | 0 | – | – | – | 0 | – | – | – |
| Without fluoropyrimidines |  |  |  |  |  |  |  |  |
| PTX + RAM | 48 (32.7) | 5.1 (4.0–6.1) | 48.7 | 8.8 (5.6–10.6) | 18 (10.7) | 5.9 (3.3–9.5) | 46.7 | 7.1 (4.0–16.2) |
| nab-PTX + RAM | 14 (9.5) | 5.0 (2.4–7.2) | 54.5 | 5.8 (2.4–19.9) | 8 (4.8) | 5.6 (1.2–17.5) | 50.0 | 10.3 (1.2–24.1) |
| PTX | 9 (6.1) | 2.8 (0.1–5.4) | 25.0 | 2.8 (0.1–6.4) | 6 (3.6) | 3.2 (1.0–9.7) | 50.0 | 12.4 (1.0–22.1) |
| nab-PTX | 4 (2.7) | 3.1 (0.1–4.7) | 0.0 | 3.1 (0.1–4.7) | 4 (2.4) | 5.8 (2.3–5.8) | 50.0 | 8.5 (5.8–11.1) |
| RAM | 4 (2.7) | 4.1 (0.1–5.0) | 75.0 | 5.8 (0.1–13.9) | 1 (0.6) | 2.6 | 100.0 | 11.1 |
| DTX | 1 (0.7) | 1.6 | 0.0 | 1.6 | 0 | – | – | – |
| IRI | 2 (1.4) | 2.4 (1.4–3.4) | 50.0 | 18.9 (1.4–36.3) | 0 | – | – | – |
| IRI + RAM | 0 | – | – | – | 0 | – | – | – |
| PEM | 0 | – | – | – | 0 | – | – | – |

*1L* first line, *2L* second line, *5-FU* 5-fluorouracil, *CapeOX* capecitabine plus oxaliplatin, *DTX* docetaxel, *DS* S-1 plus docetaxel, *FOL* folinic acid, *FOLFOX* folinic acid plus 5-fluorouracil plus oxaliplatin, *IRI* irinotecan, *NA* not available, *nab-PTX* nab-paclitaxel, *NR* not reached, *PEM* pembrolizumab, *PTX* paclitaxel, *RAM* ramucirumab, *S-1* tegafur/gimeracil/oteracil potassium, *SOX* S-1 plus oxaliplatin, *SP* S-1 plus cisplatin, *T-mab/T-DXd [–]* with no history of trastuzumab or trastuzumab deruxtecan treatment, *XP* capecitabine plus cisplatin

^a^Post-recurrent first-line therapy started ≤180 days after end of adjuvant therapy

^b^Post-recurrent first-line therapy started >180 days after end of adjuvant therapy

^c^Data presented as median (95% confidence interval)

**(b)**

|  | Early recurrence^a^ (n = 305) | | | | Late recurrence^b^ (n = 265) | | | |
| --- | --- | --- | --- | --- | --- | --- | --- | --- |
|  | Patients (%) | 1L treatment duration (months)^c^ | 1L→2L transition (%) | Overall treatment duration (months)^c^ | Patients (%) | 1L treatment duration (months)^c^ | 1L→2L transition (%) | Overall treatment duration (months)^c^ |
| Total |  | 4.6 (4.0–5.1) | 55.8 | 8.1 (7.2–10.8) |  | 4.9 (4.5–5.6) | 58.6 | 9.5 (8.1–11.0) |
| With fluoropyrimidines |  |  |  |  |  |  |  |  |
| CapeOX | 58 (19.0) | 4.2 (3.5–5.3) | 70.2 | 12.0 (7.9–19.4) | 31 (11.7) | 4.5 (2.6–6.0) | 63.3 | 7.9 (5.0–23.5) |
| SP | 22 (7.2) | 4.7 (3.3–9.5) | 80.0 | 13.0 (5.1–19.4) | 34 (12.8) | 5.1 (4.0–6.0) | 71.9 | 9.8 (6.0–12.1) |
| XP | 19 (6.2) | 2.8 (2.6–6.6) | 53.8 | 9.7 (2.7–27.9) | 8 (3.0) | 3.4 (1.9–7.9) | 50.0 | 4.0 (1.9–22.8) |
| SOX | 16 (5.2) | 4.3 (2.1–5.8) | 53.3 | 6.5 (3.5–15.6) | 79 (29.8) | 5.1 (4.0–6.2) | 62.5 | 12.0 (7.6–16.2) |
| S-1 | 7 (2.3) | 4.9 (0.5–7.2) | 85.7 | 24.8 (0.5–NR) | 33 (12.5) | 5.9 (4.2–8.5) | 46.7 | 10.4 (8.5–14.6) |
| FOLFOX | 4 (1.3) | 3.4 (1.4–7.3) | 25.0 | 5.2 (1.4–7.8) | 9 (3.4) | 4.0 (2.8–6.0) | 50.0 | NR (2.8–NR) |
| DS | 2 (0.7) | 6.9 (6.2–7.5) | 50.0 | 7.6 (6.2–9.0) | 3 (1.1) | 10.6 (0.8–10.6) | 50.0 | 10.3 (10.0–10.6) |
| FOL + 5-FU | 1 (0.3) | 1.2 | 100.0 | 3.1 | 3 (1.1) | 6.3 (0.4–15.1) | 66.7 | 18.7 (0.4–18.7) |
| FOL + 5-FU + cisplatin | 0 | – | – | – | 0 | – | – | – |
| FOL + 5-FU + PTX | 0 | – | – | – | 1 (0.4) | 3.4 | 100.0 | NR (NR–NR) |
| Without fluoropyrimidines |  |  |  |  |  |  |  |  |
| PTX + RAM | 96 (31.5) | 4.9 (4.0–6.2) | 54.4 | 7.4 (6.2–12.3) | 31 (11.7) | 4.4 (3.1–6.0) | 42.9 | 6.5 (3.7–10.9) |
| nab-PTX + RAM | 33 (10.8) | 5.0 (3.5–7.4) | 37.9 | 7.4 (4.9–15.3) | 15 (5.7) | 5.6 (3.0–10.3) | 58.3 | 8.9 (5.4–17.6) |
| PTX | 26 (8.5) | 4.6 (1.0–7.9) | 28.6 | 7.3 (1.0–11.6) | 11 (4.2) | 8.1 (1.2–11.3) | 70.0 | 11.5 (1.4–17.4) |
| nab-PTX | 10 (3.3) | 2.6 (1.0–9.4) | 42.9 | 3.9 (1.0–14.8) | 1 (0.4) | 13.6 | 100.0 | NR (NR–NR) |
| RAM | 6 (2.0) | 3.0 (0.1–7.8) | 50.0 | 7.8 (0.1–16.2) | 3 (1.1) | 2.4 (0.1–2.4) | 0.0 | 2.4 (0.1–2.4) |
| DTX | 4 (1.3) | 4.1 (1.4–5.6) | 66.7 | 7.4 (1.4–10.7) | 2 (0.8) | 19.5 (4.7–34.4) | 50.0 | 20.5 (6.6–34.4) |
| IRI | 0 | – | – | – | 1 (0.4) | – | – | – |
| IRI + RAM | 1 (0.3) | 3.5 | 0.0 | 3.5 | 0 | – | – | – |
| PEM | 0 | – | – | – | 0 | – | – | – |

*1L* first line, *2L* second line, *5-FU* 5-fluorouracil, *CapeOX* capecitabine plus oxaliplatin, *DTX* docetaxel, *DS* S-1 plus docetaxel, *FOL* folinic acid, *FOLFOX* folinic acid plus 5-fluorouracil plus oxaliplatin, *IRI* irinotecan, *NA* not available, *nab-PTX* nab-paclitaxel, *NR* not reached, *PEM* pembrolizumab, *PTX* paclitaxel, *RAM* ramucirumab, *S-1* tegafur/gimeracil/oteracil potassium, *SOX* S-1 plus oxaliplatin, *SP* S-1 plus cisplatin, *T-mab/T-DXd [–]* with no history of trastuzumab or trastuzumab deruxtecan treatment, *XP* capecitabine plus cisplatin

^a^Post-recurrent first-line therapy started ≤180 days after end of adjuvant therapy

^b^Post-recurrent first-line therapy started >180 days after end of adjuvant therapy

^c^Data presented as median (95% confidence interval)

**(c)**

|  | Early recurrence^a^ (n = 212) | | | | Late recurrence^b^ (n = 160) | | | |
| --- | --- | --- | --- | --- | --- | --- | --- | --- |
|  | Patients (%) | 1L treatment duration (months)^c^ | 1L→2L transition (%) | Overall treatment duration (months)^c^ | Patients (%) | 1L treatment duration (months)^c^ | 1L→2L transition (%) | Overall treatment duration (months)^c^ |
| Total |  | 4.4 (3.7–4.9) | 52.7 | 9.7 (7.3–12.0) |  | 4.7 (3.5–5.6) | 43.3 | 7.4 (5.6–10.0) |
| With fluoropyrimidines |  |  |  |  |  |  |  |  |
| CapeOX | 30 (14.2) | 5.2 (3.5–6.8) | 72.0 | 12.9 (9.5–20.5) | 26 (16.3) | 6.2 (3.5–8.3) | 57.1 | 10.8 (6.2–12.5) |
| SP | 18 (8.5) | 3.6 (2.4–4.4) | 47.1 | 6.7 (4.0–18.2) | 21 (13.1) | 3.5 (2.4–5.1) | 50.0 | 8.1 (2.4–18.2) |
| XP | 6 (2.8) | 4.6 (0.5–11.2) | 66.7 | 20.1 (0.5–29.5) | 1 (0.6) | 5.9 | 100.0 | 9.3 |
| SOX | 6 (2.8) | 4.7 (2.6–8.6) | 80.0 | NR (3.5–NR) | 33 (20.6) | 5.6 (3.7–7.4) | 44.4 | 8.0 (5.6–NR) |
| S-1 | 7 (3.3) | 3.0 (0.5–4.2) | 42.9 | 4.7 (0.5–32.9) | 36 (22.5) | 3.0 (2.1–4.9) | 30.0 | 4.9 (2.3–10.2) |
| FOLFOX | 2 (0.9) | 6.9 (1.0–12.8) | 100.0 | NR (NR–NR) | 3 (1.9) | 0.1 (0.1–5.9) | 33.3 | 0.1 (0.1–NR) |
| DS | 1 (0.5) | 7.9 | 100.0 | NR | 1 (0.6) | 6.0 | 0.0 | 6.0 |
| FOL + 5-FU | 0 | – | – | – | 0 | – | – | – |
| FOL + 5-FU + cisplatin | 0 | – | – | – | 0 | – | – | – |
| FOL + 5-FU + PTX | 0 | – | – | – | 0 | – | – | – |
| Without fluoropyrimidines |  |  |  |  |  |  |  |  |
| PTX + RAM | 82 (38.7) | 5.3 (4.2–7.4) | 52.9 | 10.6 (7.4–13.4) | 15 (9.4) | 8.1 (2.3–11.5) | 72.7 | 12.5 (8.8–NR) |
| nab-PTX + RAM | 20 (9.4) | 4.2 (2.6–5.4) | 50.0 | 5.4 (4.0–12.0) | 6 (3.8) | 1.4 (1.1–7.8) | 40.0 | 1.4 (1.1–NR) |
| PTX | 26 (12.3) | 3.3 (1.2–3.9) | 34.8 | 3.5 (1.2–6.1) | 10 (6.3) | 3.1 (0.5–6.2) | 33.3 | 3.1 (0.5–7.1) |
| nab-PTX | 7 (3.3) | 2.8 (0.1–7.0) | 42.9 | 9.3 (0.1–21.6) | 4 (2.5) | 2.0 (1.4–7.2) | 25.0 | 2.0 (1.4–13.9) |
| RAM | 5 (2.4) | 5.3 (0.1–16.6) | 0.0 | 5.3 (0.1–16.6) | 1 (0.6) | 3.3 | 0.0 | 3.3 |
| DTX | 1 (0.5) | 4.2 | 100.0 | NR | 1 (0.6) | 4.9 | 0.0 | 4.9 |
| IRI | 1 (0.5) | 8.8 | 100.0 | 13.7 | 1 (0.6) | 2.6 | 0.0 | 2.6 |
| IRI + RAM | 0 | – | – | – | 0 | – | – | – |
| PEM | 0 | – | – | – | 1 (0.6) | 0.1 | 0.0 | 0.1 |

*1L* first line, *2L* second line, *5-FU* 5-fluorouracil, *CapeOX* capecitabine plus oxaliplatin, *DTX* docetaxel, *DS* S-1 plus docetaxel, *FOL* folinic acid, *FOLFOX* folinic acid plus 5-fluorouracil plus oxaliplatin, *IRI* irinotecan, *NA* not available, *nab-PTX* nab-paclitaxel, *NR* not reached, *PEM* pembrolizumab, *PTX* paclitaxel, *RAM* ramucirumab, *S-1* tegafur/gimeracil/oteracil potassium, *SOX* S-1 plus oxaliplatin, *SP* S-1 plus cisplatin, *T-mab/T-DXd [–]* with no history of trastuzumab or trastuzumab deruxtecan treatment, *XP* capecitabine plus cisplatin

^a^Post-recurrent first-line therapy started ≤180 days after end of adjuvant therapy

^b^Post-recurrent first-line therapy started >180 days after end of adjuvant therapy

^c^Data presented as median (95% confidence interval)

## Supplementary Table S9

Post-recurrent first-line treatment patterns in the T-mab/T-DXd [+] group of the recurrent cohort

|  | Early recurrence^a^ (n = 89) | | | | Late recurrence^b^ (n = 34) | | | |
| --- | --- | --- | --- | --- | --- | --- | --- | --- |
|  | Patients (%) | 1L treatment duration (months)^c^ | 1L→2L transition (%) | Overall treatment duration (months)^c^ | Patients (%) | 1L treatment duration (months)^c^ | 1L→2L transition (%) | Overall treatment duration (months)^c^ |
| Total |  | 5.8 (5.2–7.7) | 61.9 | 16.6 (12.2–21.9) |  | 7.8 (5.3–9.3) | 71.9 | 16.1 (8.8–21.4) |
| T-mab–based regimens |  |  |  |  |  |  |  |  |
| With fluoropyrimidines |  |  |  |  |  |  |  |  |
| XP + T-mab | 44 (49.4) | 7.9 (5.8–9.6) | 54.8 | 18.0 (11.1–36.7) | 14 (41.2) | 9.4 (3.3–18.4) | 78.6 | 18.5 (7.2–50.9) |
| CapeOX + T-mab | 15 (16.9) | 5.6 (4.0–9.7) | 60.0 | 12.2 (5.3–26.9) | 3 (8.8) | 7.5 (7.3–7.8) | 100.0 | 22.0 (13.7–30.2) |
| SOX + T-mab | 3 (3.4) | 5.4 (0.3–18.2) | 66.7 | 0.3 | 3 (8.8) | 7.7 (5.3–10.2) | 50.0 | 11.0 (10.2–11.8) |
| SP + T-mab | 1 (1.1) | 4.6 | 100.0 | NA^d^ | 4 (11.8) | 6.5 (1.0–16.0) | 75.0 | 21.0 (1.0–24.7) |
| Without fluoropyrimidines |  |  |  |  |  |  |  |  |
| PTX + T-mab | 9 (10.1) | 8.2 (2.8–39.9) | 83.3 | 42.3 (6.0–42.3) | 3 (8.8) | 4.4 (4.2–9.3) | 100.0 | 11.8 (5.8–16.1) |
| IRI + T-mab | 1 (1.1) | 9.7 | 100.0 | 15.7 | 0 | – | – | – |
| non-T-mab–based regimens |  |  |  |  |  |  |  |  |
| With fluoropyrimidines |  |  |  |  |  |  |  |  |
| SP | 3 (3.4) | 3.5 (2.3–4.0) | 66.7 | 19.4 (4.0–19.4) | 1 (2.9) | 3.5 | 0.0 | 3.5 |
| CapeOX | 2 (2.3) | 2.7 (2.2–3.2) | 100.0 | 39.1 | 1 (2.9) | 8.0 | 0.0 | 8.0 |
| SOX | 1 (1.1) | 5.0 | 100.0 | 24.6 | 1 (2.9) | 5.6 | 100.0 | 35.4 |
| S-1 | 1 (1.1) | 2.1 | 0.0 | 2.1 | 2 (5.9) | 7.1 | 50.0 | – |
| Without fluoropyrimidines |  |  |  |  |  |  |  |  |
| nab-PTX + RAM | 4 (4.5) | 6.4 (4.2–9.3) | 75.0 | NR (7.8–NR) | 1 (2.9) | 7.2 | 100.0 | 8.8 |
| PTX + RAM | 3 (3.4) | 4.9 (3.0–5.2) | 66.7 | 13.4 (4.9– NR) | 1 (2.9) | 5.8 | 0.0 | 5.8 |
| nab-PTX | 1 (1.1) | 2.1 | 0.0 | 2.1 | 0 | – | – | – |
| PTX | 1 (1.1) | 2.8 | 100.0 | 13.4 | 0 | – | – | – |

*1L* first line, *2L* second line, *CapeOX* capecitabine plus oxaliplatin, *IRI* irinotecan, *NA* not available, *nab-PTX* nap-paclitaxel, *NR* not reached, *PTX* paclitaxel, *RAM* ramucirumab, *S-1* tegafur/gimeracil/oteracil potassium, *SOX* S-1 plus oxaliplatin, *SP* S-1 plus cisplatin, *T-mab* trastuzumab, *T-mab/T-DXd [+]* with history of trastuzumab or trastuzumab deruxtecan treatment, *XP* capecitabine plus cisplatin

^a^Post-recurrent 1L therapy started ≤180 days after end of adjuvant therapy

^b^Post-recurrent 1L therapy started >180 days after end of adjuvant therapy

^c^Data presented as median (95% confidence interval)

^d^Patient was censored after 2L therapy start

## Supplementary Fig. S1

Study design


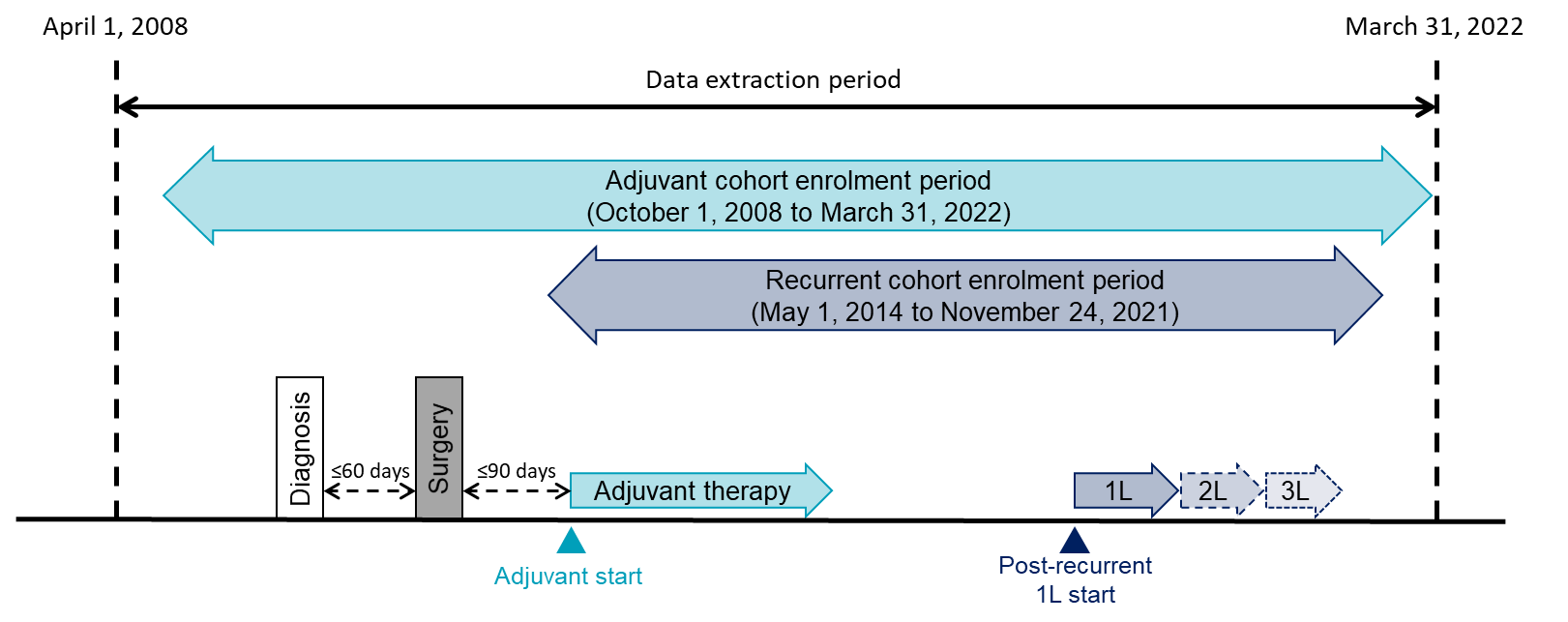


*1L* first line, *2L* second line, *3L* third line

## Supplementary Fig. S2

Treatment sequence in T-mab/T-DXd [+] group of the recurrent cohort with **(a)** early recurrence (≤180 days from end of adjuvant therapy) or **(b)** late recurrence (>180 days from end of adjuvant therapy)


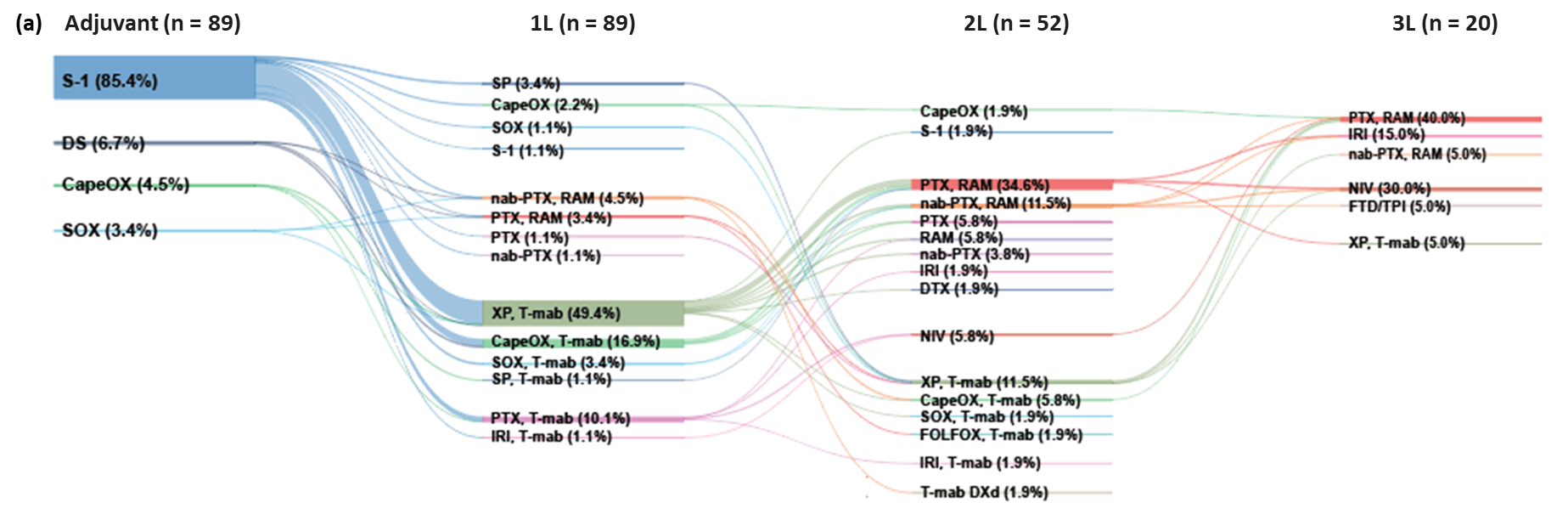

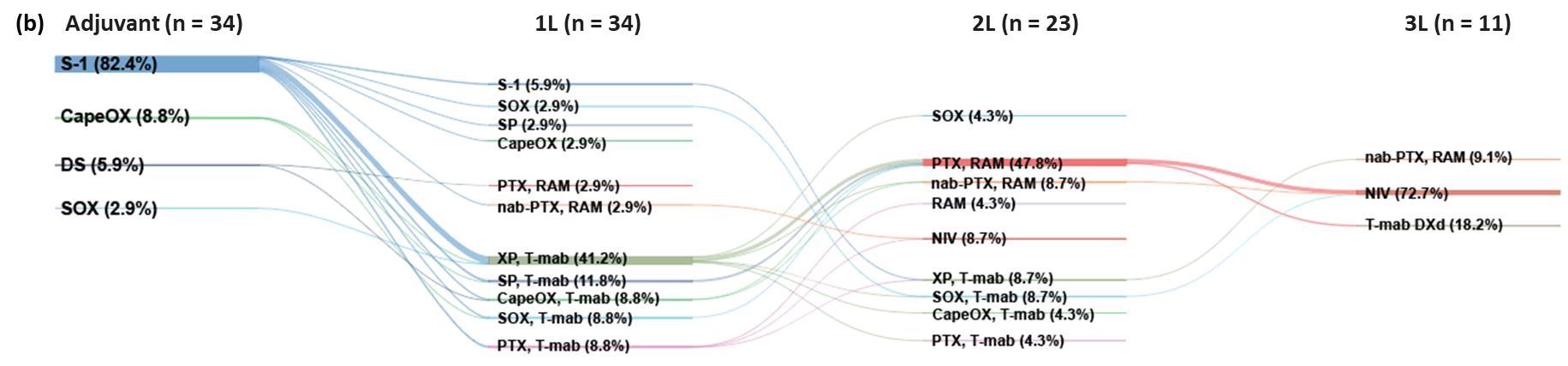


*1L* first line, *2L* second line, *3L* third line, *CapeOX* capecitabine plus oxaliplatin, *DS* S-1 plus docetaxel, *DTX* docetaxel, *FTD/TPI* trifluridine/tipiracil, *FOLFOX* folinic acid plus 5-fluorouracil plus oxaliplatin, *HER2* human epidermal growth factor receptor 2, *IRI* irinotecan, *nab-PTX* nab-paclitaxel, *NIV* nivolumab, *PTX* paclitaxel, *RAM* ramucirumab, *S-1* tegafur/gimeracil/oteracil potassium, *SOX* S-1 plus oxaliplatin, *SP* S-1 plus cisplatin, *T-mab* trastuzumab, *T-mab DXd* trastuzumab deruxtecan, *T-mab/T-DXd [+]* with a history of T-mab or T-mab DXd treatment, *XP* capecitabine plus cisplatin
